# Impacts of strigolactone on shoot branching under phosphate starvation in chrysanthemum (*Dendranthema grandiflorum* cv. *Jinba*)

**DOI:** 10.3389/fpls.2015.00694

**Published:** 2015-09-11

**Authors:** Lin Xi, Chao Wen, Shuang Fang, Xiaoli Chen, Jing Nie, JinFang Chu, Cunquan Yuan, Cunyu Yan, Nan Ma, Liangjun Zhao

**Affiliations:** ^1^Beijing Key Laboratory of Development and Quality Control of Ornamental Crops, Department of Ornamental Horticulture and Landscape Architecture, China Agricultural UniversityBeijing, China; ^2^National Centre for Plant Gene Research, Institute of Genetics and Developmental Biology, Chinese Academy of SciencesBeijing, China

**Keywords:** auxin, chrysanthemum bud outgrowth, local regulation, phosphorus starvation, strigolactone (SL), systemic regulation, UPLC-MS

## Abstract

Chrysanthemum (*Dendranthema grandiflorum cv. Jinba*) shoot branching is determined by bud outgrowth during the vegetative growth stage. The degree of axillary bud outgrowth is highly influenced by environmental conditions, such as nutrient availability. Here, we demonstrated that phosphorus (Pi) starvation significantly reduces axillary bud outgrowth in chrysanthemum. A strigolactone (SL) biosynthesis gene, *DgCCD7*, was isolated and characterized as an ortholog of *MAX3/DAD3/RMS5/D17*. By using ultra-performance liquid chromatography coupled with mass spectrometry (UPLC-MS), three putative SLs were identified and levels of all three SLs showed strong increase under Pi starvation conditions. Determinations of the distribution of SLs and regulation of *DgCCD7/8* in response to Pi changes in root indicate that SL acts systemically. However, temporal expression patterns of biosynthesis and signaling genes in nodes revealed that Pi starvation causes a local response of SL pathway. Treatment of node segments with or without auxin and Pi revealed that in the absence of exogenous auxin, Pi delayed axillary buds outgrowth and up-regulated local SL pathway genes. These data indicated that an auxin-SL regulatory loop responded to Pi starvation for delaying bud outgrowth locally, root biosynthesized SLs were transported acropetally and functioned in shoot branching inhibition under Pi starvation. We proposed that SLs contributed to chrysanthemum shoot branching control in response to Pi-limiting conditions in a systemic way.

## Introduction

Shoot branching plays an important role in establishing plant architecture during development and growth, and also confers flexibility of response to environmental stress in plants. In ornamental horticulture, shoot branching is directly associated with ornamental value and industrial economics. Chrysanthemum (*Dendranthema grandiflorum cv. Jinba*) is one of the major cut flowers cultivated for domestic use worldwide. In China, Japan and Korea, standard commercial cut chrysanthemums are required to bear a large flower on each stem. Manual pinching or removal of lateral buds and branches is a necessary procedure during the vegetative growth stage, which comprises one-third of the production cost. Thus, clarification of the mechanisms underlying shoot branching, and effective approaches to control the process are essential for both breeders and farmers.

Previous studies have shown that axillary buds in chrysanthemum plants form in advance and remain dormant. Outgrowth is associated with bud position (Deruiter, 1996; Chen et al., 2013). Apical dominance, a well-known phenomenon whereby basally located axillary buds on the main stem are suppressed by apical tissues, plays an important part in controlling bud outgrowth in chrysanthemum. Studies on plant models have shown that auxin inhibits bud outgrowth indirectly by its regulation of strigolactone (SL), an acropetally transported hormone mainly synthesized in root (Brewer et al., 2009, 2013).

Strigolactone and its derived metabolite(s) were previously identified as chemicals in root exudates in the rhizosphere that promotes seed germination of parasitic plants, Striga and Orobanche (Cook et al., 1966; Yokota et al., 1998). Increased branching mutants in rice (*Oryza sativa*), pea (*Pisum sativum*), and Arabidopsis and their root exudates showed a decreased capacity to stimulate parasitic weeds and lack of SLs (Gomez-Roldan et al., 2008; Umehara et al., 2008). Several steps in SL biosynthesis and related signaling pathways have been clarified. SL is biosynthesized from isomerization of all-*trans* β-carotene to 9-*cis*-β-carotene by isomerases, D27 (Lin et al., 2009; Alder et al., 2012; Waters et al., 2012). Carotenoid cleavage dioxygenase 7 (CCD7) cleaves at the C9′-C10′ position to generate 9-*cis*-β-apo-10′-carotenal substrate, to which carotenoid cleavage dioxygenase 8 (CCD8) incorporates three added oxygens to form the A–ring, characteristic D–ring and enol-ether bridge (Alder et al., 2012; Waldie et al., 2014). Then the product, which is named carlactone, is modified by cytochrome P450 MAX1 and other unidentified components to generate active SLs (Booker et al., 2005). It is reported that two members of rice MAX1 homologous enzymes act either as a carlactone (CL) oxidase to stereoselectively convert CL into ent-2′-*epi*-5-deoxystrigol (*epi*-5DS) or catalyzes the subsequent hydroxylation of the latter to produce orobanchol (Zhang et al., 2014). D14 (Petunia DAD2), an α/β–hydrolase has been shown to participate in an SL–sensitive receptor-degradation/ubiquitination complex together with MAX2/D3/RMS4 (Gaiji et al., 2012; Hamiaux et al., 2012; Zhou et al., 2013), another component integral to SL perception and signaling (Stirnberg et al., 2007). Comparative studies have recently been extended to other species (Vogel et al., 2010; Guan et al., 2012; Dong et al., 2013; Ward et al., 2013; Rubio-Moraga et al., 2014).

The most common method used to identify SLs is high performance liquid chromatography/tandem mass spectrometry (LC-MS/MS), which can also be effectively applied for quantifying the SL concentrations in plants under the multiple reaction monitoring (MRM) mode with ultra-high sensitivity (Sato et al., 2003). With the aid of this method, SLs have been successfully identified and quantified in Arabidopsis, rice, pea and tomato (Gomez-Roldan et al., 2008; López-Ráez et al., 2008; Umehara et al., 2008; Kohlen et al., 2011). Based on the identified SL and its analogs, an ultra-performance liquid chromatography (UPLC)-MS screening method was fully established to detect potential SL analogs. This method has been applied for the quantification of *epi*-5DS in rice (Lin et al., 2009; Zhou et al., 2013). However, UPLC-MS can be combined with germination assays using seeds of root parasitic plants, e.g., *Orobanche minor*, for verifying known or unknown SLs. In *Asteraceae* plants, seven known SLs have been detected using LC-MS/MS but only major germination stimulants may be detected in germination assays for normal plant culture systems (Yoneyama et al., 2011). Much evidence has shown that SLs accumulation would increase with phosphate deficiency in root exudates (López-Ráez et al., 2008; Kohlen et al., 2011). Therefore, using UPLC-MS screening to identify potential SLs, combined with the quantification of their increase under phosphate limiting conditions, can be of help for fast-verification of SLs and their analogs in horticultural plants.

Over the last decade, controversial data have been obtained for the SL regulation mechanisms, mainly focusing on the relationship with auxin. One hypothesis highlights root synthesized SLs tend to move acropetally to act as a long-distance secondary messenger for auxin in direct controlling bud outgrowth (Brewer et al., 2009; Hayward et al., 2009). Since BRANCHED 1 (BRC1) transcription factor proved to be an integrator of branching signals within axillary buds (Aguilar-Martinez et al., 2007; Koyama et al., 2007), it is also proposed that BRC1 is required for SL direct inhibition of buds' outgrowth (Dun et al., 2012, 2013; Brewer et al., 2013). Recent evidence revealed that SLs act independently of auxin transport/canalization to inhibit bud outgrowth in pea (Brewer et al., 2015), which strongly supported this hypothesis. Another hypothesis, auxin transport/canalization hypothesis, supports the mechanism that SLs perform indirect inhibition of shoot branching by regulating auxin transportation, which is in need of the presence of a competing auxin source (Ongaro and Leyser, 2008; Crawford et al., 2010; Liang et al., 2010; Balla et al., 2011; Ward et al., 2013). In this case, auxin in the main stem is thought to act by preventing the flow of auxin out from buds, which leads to buds inhibition (Prusinkiewicz et al., 2009). This systematic regulation mechanism was further confirmed with the detection of PIN-FORMED1 (PIN1) protein reduction in xylem parenchyma cells, together with auxin transport suppression (Shinohara et al., 2013). Computational modeling studies indicated that PIN1 depletion from the plasma membrane of xylem parenchyma cells in the stem is triggered by SL signaling, and is independent of protein synthesis (Prusinkiewicz et al., 2009; Shinohara et al., 2013). Transcription of SL biosynthetic genes can be up-regulated by auxin as well, which indicate the feed-back regulation between auxin and SLs (Foo et al., 2005; Zou et al., 2006; Arite et al., 2007; Hayward et al., 2009; Liang et al., 2010). Thus, SLs participate in the systemic and local regulation of shoot branching to balance growth across the shoot system.

As well as the roles described previously, SLs also have a role in the development of the root system architecture (Koltai, 2013). They inhibit lateral root growth under high phosphate conditions (Kapulnik et al., 2011), and enhance their formation under low Pi conditions (Ruyter-Spira et al., 2011). This role suggested that SLs play an important part in nutrient uptake for plants. Under low nitrogen condition, SL signaling participated in delaying of axillary buds activation coupled with auxin (de Jong et al., 2014). In petunia, gene expression of SL pathway responds to light quality and Pi status (Drummond et al., 2015). Meanwhile SL pathway contributed to root response to Pi and nitrogen deficiency (Sun et al., 2014). Considerable evidence has confirmed a positive correlation between inorganic phosphate (Pi) starvation and SL biosynthesis (López-Ráez et al., 2008; Domagalska and Leyser, 2011; Kohlen et al., 2011). In chrysanthemum, physiology research on phosphate nutrient starvation confirmed changes of root architecture by phosphate deficiency, but less has been described about the influence on shoot branching (Laishram et al., 2013). Similarly to the roles described above, SLs have also been shown to participate in shoot branching reduction in rice and Arabidopsis (Umehara et al., 2010; Kohlen et al., 2011; Puig et al., 2012). Therefore, in the current study, a UPLC-electrospray ionization (ESI)-Qtrap-MS screening method was applied to determine SLs analogs in chrysanthemum which could lead to the identification of SL derivatives in horticultural plants. A further investigation was carried out on the impact of Pi absence on the SL pathway gene expression. This result gave a hint that in chrysanthemum, the reasonable control of nutrient (Pi) supply would benefit buds' inhibition at the earlier stage of vegetative growth.

## Materials and methods

### Plant materials, Pi starvation treatment, growth conditions and bud length measurement

**Figure 1:**
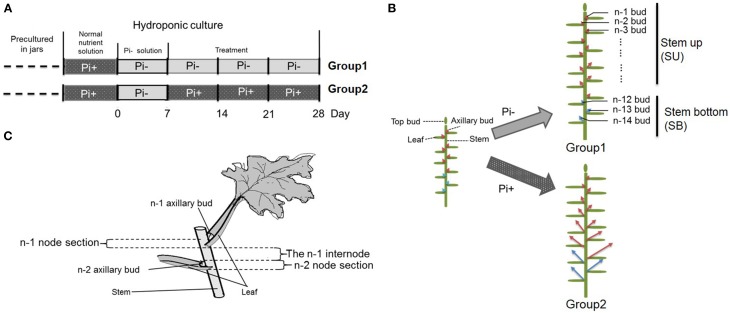
**Schematic diagram showing the experimental conditions**. **(A)** Four week-old seedlings grown on agar medium in jars were transferred to hydroponic normal culture medium for 1 week then changed into solution without Pi for another week, followed by culture in medium with continued Pi starvation or containing normal concentrations of Pi. **(B)** Overall schematic of chrysanthemum branching status before and after treatment. Arrows mark axillary buds. Red arrows represent uniform shoots which were used in whole experiment; Blue arrows represent basal shoots which were from propagated cuttings. The definition of buds in different position along with main stem is shown at right side. n–1 bud means the very apical axillary bud. Stem up (SU) and Stem bottom (SB) position are notified in the schematic. **(C)** The definition of node/internode section. The n–1 and n–2 sections are presented.

Plantlets of chrysanthemum (*Dendranthema grandiflorum* cv. *Jinba*) were propagated under sterile conditions in jars with Murashige and Skoog (MS) agar medium (Murashige and Skoog, 1962), followed by growth in a tissue culture room at 24°C/18°C under a photoperiod of 16 h/8 h light/dark and light intensity of ~100–120 μmol photons·m^−2^·s^−1^. For chrysanthemum hydroponics, 4-week-old plantlets were transferred out of sterile conditions into flasks containing Hoagland nutrient solution (Tocquin et al., 2003). Plants were cultured hydroponically for 1 week in Hoagland nutrient solution with 1 mM monopotassium phosphate (KH_2_PO_4_). Then the plants were transferred into low phosphate conditions containing 2 μM KH_2_PO_4_ in culture medium. Potassium (K^+^) was added to equal the amount present in the sufficient-phosphate conditions to exclude potential effects of K^+^ on the treatments by using K_2_SO_4_. Later plants were separated into two groups. Group one contained plants cultured in Pi-depleted conditions and Group two contained plants cultured in parallel with fully fertilized nutrient (Figures 1A,B). For an experiment over time, the roots of 20 plants/per group were harvested at each time point after group separation. The harvesting began from the time point marked with a red triangle (Figure 1A). For the temporal Pi starvation/resupply experiment, upper node sections of the plants (without leaf) were harvested after being subjected to Pi starvation at day 0 and resupplied with Pi at day 7. The plant materials were collected beginning from each time point to 48 h after. The nutrient solution was replaced twice a week. The outgrowth of lateral buds was recorded 28 days after the plants were replaced in hydroponic culture. For convenience, the lateral buds were numbered basipetally as n-1, n-2 (Figure 1B).

Plants were grown under controlled conditions in a climate chamber under 16 h light/8 h dark at 24°C/18°C and light intensity of 120 μmol photons·m^−2^·s^−1^. Seeds of *Arabidopsis thaliana max3-9* and *Columbia-0* lines were sterilized in 2% sodium hypochlorite containing 0.02% (*v/v*) Tween-20, rinsed thoroughly with sterile water, and stratified for 3 days at 4°C in the dark. After germination on ½MS agar medium, seedlings were transferred in to pots (9 cm × 9 cm) containing peat soil and vermiculite (1:1) in the climate chamber under 16 h light/8 h dark at 24°C/18°C and a light intensity of 120 μmol photons ·m^−2^·s^−1^.

An image of each axillary bud was captured on to graph paper (1 mm^2^ for a single square). Then the image was imported into ImageJ 1.49. The image of the bud can be magnified and the length of the bud can be calculated.

### Quantification of indole acetic acid using enzyme-linked immunosorbent assay (ELISA)

Freeze dried stem samples from different positions of the internodes were ground into fine powder and dissolved in 2.0 ml of phosphate buffered saline (PBS) containing 0.1% (*v/v*) Tween-20 and 0.1% (*w/v*) gelatin (pH 7.5) to quantify the amount of free indole acetic acid (IAA) using ELISA following a protocol described previously (Zhao et al., 2006). The mouse monoclonal antibodies against free IAA were produced at the Center of Crop Chemical Control, China Agricultural University, China (Weiler et al., 1981; Wang et al., 2012). The ELISA data were calculated as described previously in Weiler et al. (1981). The relative IAA concentration was calculated using the IAA concentration [ng/g fresh weight (FW)] detected where the stem sections join divided by the IAA concentration (ng/g FW) in the top shoots.

### Root exudate collection and UPLC-ESI-Qtrap-MS

Each hydroponic cultured chrysanthemum plant was placed in 100 ml of nutrient solution within an aluminum foil covered flask to exclude light. The medium was changed every 3 days. On the day of root exudate collection, chrysanthemum roots were washed with tap water and returned to new nutrient solutions (50 ml per plant) for 5 h. The nutrient solutions were then collected. Meanwhile, 30 plants were harvested and separated into three parts: top shoot, basal shoot and roots. The root FW of each plantlet was determined. All materials were immediately immersed in liquid nitrogen and stored at −80°C. The deuterated internal standard (D_6_-epi-5DS, The University of Tokyo) was added before the purification step in the determination of SL. From the plant tissue, the SL was purified sequentially using Sep-Pak silica (WAT043400, Waters) and Oasis HLB (WAT094226, Waters) solid-phase extraction columns. From the root exudate, purification of SLs was achieved using only the Oasis HLB column. The final purified samples were eluted by acetone and re-dissolved in acetonitrile (ACN).

SL detection was performed using UPLC (Waters, Milford, MA, USA) combined with a 5500 Qtrap MS equipped with an ESI source (AB Sciex, Foster City, CA, USA). Odd mass-to-charge precursors were selected from *m/z* 299 to *m/z* 425 for each MRM transition for screening of SLs and their analogs (Gomez-Roldan et al., 2008; Yoneyama et al., 2011). As the D-ring and enol-ether linkage to the tricyclic lactone (ABC rings) are essential moieties for maintaining bioactivity, the fragment ion of the D-ring, *m/z* 97, was selected as the diagnostic daughter ion for each MRM transition. The information dependent acquisition (IDA) of collision induced dissociation (CID) MS/MS spectrum was triggered by setting the threshold of MRM transition intensity above 1000 cps to qualify potential SLs. The UPLC and MS parameter settings were: ionization: 5500 V, temperature: 550°C, curtain gas: 35 psi, ion source gas (GS1): 50 psi, and turbogas (GS2): 50 psi. The dynamic fill time option was selected to monitor each MRM-IDA-CID cycle. In this operation mode, the scan rate was 10,000 Da/s and CE 30 ± 10 V. Root extracts of chrysanthemum were analyzed using UPLC with a ethylene bridged hybrid column (BEH) C_18_ column (2.1 mm × 100 mm, 1.7 μm). Mobile phases A and B were HPLC-grade water and ACN containing 0.05% acetic acid, respectively. A linear gradient was set from 50% B to 90% A in 5 min, increased to 98% after 0.5 min, and maintained at 98% B for 1.5 min. The flow rate was 300 μL/min and the injection volume was 5 μL.

### RNA extraction and gene isolation

Total RNA was extracted from chrysanthemum using TRIzol Reagent (Invitrogen, 15596-026). cDNA was synthesized using SuperScript III reverse transcriptase (Invitrogen, 18080-044). Degenerate primers were designed based on a sequence alignment of *CCD7* from different species (Supplementary Table S3). A fragment of the partial *DgCCD7* gene was obtained, which was extended using Rapid Amplification of cDNA Ends (RACE) PCR. Amplified fragments were cloned into the pMD18-T vector (Takara, D101A) and sequenced. Genomic DNA was isolated from young tissue using the SurePlant DNA Kit (Cwbio, CW0555). Amplified products were used to determine genomic clones and intron positions in *DgCCD7* genes.

### Gene expression analysis

Total RNA was extracted using TRIzol Reagent (Invitrogen, 15596-026) and quantified using NanoDrop2000. A 2 μg aliquot of total RNA was used for cDNA generation in a reaction volume of 20 μl using a FastQuant RT Kit (with gDNase) (Tiangen, KR106). All the primers used in this study are listed in Supplementary Table S3. QRT-PCR was performed in a 10 μl reaction volume with the KAPA™ SYBR® FAST qPCR kit (KapaBiosystems, Woburn, MA) on a StepOnePlus Real-Time PCR System (Applied Biosystems, Foster City, USA) according to the manufacturer's instructions. PCR products were verified via melting curve analysis. A standard curve for each target gene and control genes was generated using a dilution series of known concentrations of plasmid vectors containing target genes and measured by the same quantitative PCR process. The control genes were validated stable expression across different treatment in this experiment. Gene expression levels were normalized to 18S and ACTIN transcripts using the comparative ΔΔCt method (Livak and Schmittgen, 2001).

### Subcellular localization

To generate35S: DgCCD7-GFP plasmids, ORFs of DgCCD7a and DgCCD7b were cloned into the binary vector, pEZS-NL. As the control, pEZS-NL vector with 35S::GFP was used. For introduction into onion epidermis cells, we used biolistic transformation, as described previously (Varagona et al., 1992). After bombardment, onion peels were incubated with MS medium for 22 h at 23°C in the dark. Fluorescence signals were observed under an Eclipse C1si confocal microscope (Nikon) and images acquired using EZ-C1FreeViewer software (Nikon). The excitation wavelength was 488 nm with band pass 510–525 nm emission filters. The same vectors were introduced into protoplasts prepared from mature leaves of 5 week-old *Arabidopsis* plants using a previously described protocol (Yoo et al., 2007). After overnight incubation in the dark, GFP signal and chlorophyll autofluorescence were examined under a confocal microscope at excitation wavelengths of 488 and 647 nm, respectively (LSM510, Carl Zeiss). Images were acquired using a Zeiss LSM Image Browser.

### CCD7 activity assay

The pGEX4, pGEX4::DgCCD7a and pGEX4::DgCCD7b constructs were transformed into chemically competent BL21-DE3 (Transgen Biotech) cells harboring the pAC-BETA plasmid encoding carotenoid biosynthetic genes for producing ß-carotene (Cunningham et al., 1994). Protein expression and ß-carotene analysis were performed as described previously (Schwartz et al., 2004).

### Generation of transgenic plants

For complementation experiments, ORFs of DgCCD7a and DgCCD7b were individually fused with the 35S promoter in the binary vector pCAMBIA 1301. The constructs were transformed into *Arabidopsis thaliana max3-9* background via the *Agrobacterium tumefaciens* strain GV3101, using the floral dip method (Clough and Bent, 1998). Transformants were selected on agar-solidified MS medium containing 80 mg·L^−1^ hygromycin (Amresco, 0408). For each construct, at least 12 independent single insertion lines were taken to homozygosity.

### Shoot branching assay

To quantify branching, a decapitation assay was used (Greb et al., 2003). Seeds of *max3-9, Columbia-0* and transgenic lines were sown on ½MS agar medium for 6 days and transferred into composted soil at 21°C under an 8 h/16 h light/dark photoperiod. After 28 days, plants were shifted to a 16 h/8 h light/dark photoperiod to induce flowering. Primary bolts were decapitated once they reached 10–15 cm in height. Rosette branches were counted 10 days after decapitation.

### Split-plate and node section assay

The split-plate system was modified according to earlier studies on Arabidopsis (Chatfield et al., 2000). Fresh stock solutions of hormones were made every month and stored at −20°C. 1-Naphthaleneacetic acid (NAA) (Sigma N0640) was dissolved in 70% ethanol. The split-plate was generated as reported previously (Chen et al., 2013).

For Pi starvation, MS medium without Pi was used. After solidification of the MS full nutrient (containing 1 mM Pi) agar medium, half of the plate was cut out. The empty half plate was filled with MS agar medium without Pi. Following solidification, a split gap was made in the middle of the plate.

For single bud outgrowth activity measurement, an MS full nutrient plate was used. A split air gap was also made after medium solidification. A single bud-node section was inserted in the split-plate, and the length of bud was measured every day.

A modified node section system was used in +Pi/−Pi treatment only. Sections with apical tissues (node n-1~node n-4) were cut from chrysanthemum plantlets, the bottom of the plant was attached to MS medium for rooting. Five days later, top buds, node n-1 and node n-2 were removed; this left rooted sections with two buds (node-3 and node-4), which were inserted into the prepared media (only the root was attached to the bottom of the medium) (see Supplementary Figure S1). Petri dishes were held vertically in the room where intact plants were cultured. For each treatment, eight plants were measured for growth of lateral branches every 24 h for 10 days by aligning a ruler behind the plates. For MAX gene transcript expression analysis, both nodes and internodes were harvested separately at 4 h after treatment. All the experiments were repeated a minimum of four times.

### Statistics

Analysis of variance (ANOVA) was used followed by a Duncan's test (for more than two comparisons) or a *t*-test (two comparisons) was used (SPSS 18.0) where differences between means were assessed and their significance was determined α = 0.05.

## Results

### Pi starvation causes branching reduction and IAA relocation in chrysanthemum

**Figure 2:**
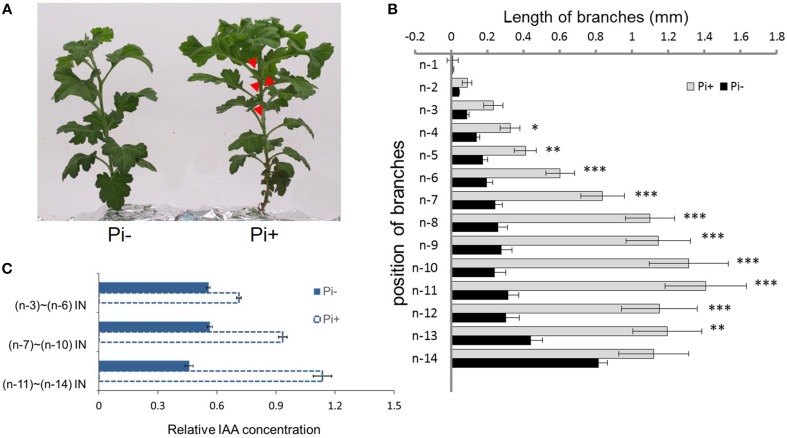
**Effect of phosphate on chrysanthemum axillary bud outgrowth and auxin status along the main stem. (A)** Twenty-eight day-old chrysanthemum plants grown under Pi starvation (left) and Pi sufficient conditions (right). Arrowheads highlight obviously branches. **(B)** Lengths of lateral branches at different node positions in 2 day-old chrysanthemum plants grown with or without 1 mM Pi. Branch positions were recorded acropetally. Bars indicate an average of 30 independent replicates ± SE. ^*^indicates significant differences at 0.05 (^*^), 0.01 (^**^), and 0.001(^***^) levels of probability. **(C)** Comparison of the levels of relative free IAA along the main stem in Pi starvation and Pi sufficient conditions. Bars indicate an average of 30 independent replicates ± SE. IN, internode. Branch positions were recorded acropetally.

We used hydroponically grown plantlets taken from cuttings and tested them under different Pi treatments. The experimental design is shown schematically in Figure 1. Both groups were treated with Pi starvation for 1 week. This was continued for group one, whereas group two was given 1 mM Pi in its hydroponic nutrient (Figure 1A). The definition of the positions of node/internode and the effect of the treatment are also shown in the diagram (Figures 1B,C). The pattern of bud length distribution (14 axillary buds) revealed that axillary buds of the whole plant were strongly inhibited upon depletion of Pi (Figures 1B, 2A,B). Under fully fertilized nutrient conditions, bud outgrowth was shown to be associated with the distance from the shoot apex from node position n-1 to n-11. A similar pattern was observed under Pi starvation culture conditions, indicating that distance to the top of the plant is one of the factors influencing bud outgrowth in chrysanthemum, at least for top 11 buds in this case. However, it was also observed that buds at the lower positions (n-12 to n-14) originated from propagated cuttings and showed less dominance from the top buds under both circumstances. Thus, the development status of buds influence bud outgrowth, which was observed in other species as well (McSteen and Leyser, 2005). As to the top three axillary buds, their outgrowth under Pi starvation displayed no significant inhibition, in comparison with buds at the same position on fully fertilized plants, and the buds below appeared more irregularly active under Pi starvation conditions.

For the architecture above the n-11 node showed a tendency to apical dominance. Internodes, n-3 to n-6 were pooled as one sample, n-7 to n-10 as another sample, and n-11 to n-14 as the remainder to detect the relative concentration of IAA along the main stem. Under Pi-containing culture conditions, a gradient distribution was observed. However, under Pi starvation, along the main stem, relative IAA concentrations remained at approximately similar levels (Figure 2C). These data suggest that IAA distribution along the main stem is altered by Pi starvation in chrysanthemum. Additionally, Pi starvation was lately shown to strongly increase endogenous SLs in peas, Arabidopsis, tomatoes and rice, which contributed to the inhibition of rice tillering and Arabidopsis branching (Umehara et al., 2010; Kohlen et al., 2011). In view of these findings, we decided to determine whether the SL function inhibits chrysanthemum shoot branching under Pi starvation.

### Pi starvation increases endogenous SL levels in chrysanthemum

To further confirm whether Pi starvation influences SL levels, which contribute to the inhibition of bud outgrowth, SL analogs in chrysanthemum (*Dendranthema grandiflorum* cv. *Jinba*) were detected using the UPLC-ESI-Qtrap-MS screening method. Three putative SL analogs were obtained in root exudates using the previously described screening strategy. According to the *m/z* of [M+H]^+^, these were denoted as 361 (retention time (RT) = 2.15 min), 363 (RT = 2.56 min), and 377 (RT = 2.52 min) (Supplementary Figures S2A,B). MS/MS spectra of the three putative SL analogs were acquired using high resolution quadrupole time-of-flight (Q-TOF) MS for confirming their structures (Supplementary Figure S2B). The diagnostic daughter ions were established by comparing them with the theoretical mass-to-charge ratio (Supplementary Table S1). The basic features of SL structure were confirmed by the presence of the fragment ion of the D-ring, *m/z* 97, a high abundance of the typical ion [M+H-CH_3_OH]^+^, and daughter ions from the loss of CH_3_OH combined with the D-ring and H_2_O/CO. Based on these results, it was concluded that all the SLs were methoxylated. Compound 361 could be dissociated into radical daughter ions of [M+H-D] ^+^ ∙ *m/z* 264.2 and [M+H-D-CH_3_OH-H_2_O]^+^ ∙ *m/z* 214.1, caused by loss of the D-ring or neutral loss of the D-ring with H_2_O/CO, which typically occurs in SLs, such as *epi-5DS*. The molecular element compositions were M_361_ = C_20_H_24_O_6_(Da), M_363_ = C_20_H_26_O_6_ (Da), and M_377_ = C_20_H_24_O_7_ (Da) (Table 1).

**Table 1:** **Accurate masses calculation of 3 putative chrysanthemum (***Dendranthema grandiflorum cv. Jinba***) SL analogs**.

**Candidate SL analogs**	**Measured m/z**	**Theoretical m/z**	**Relative error(p.p.m)**	**Molecular Formula**
361	361.1642	361.1651	2.5	C_20_H_24_O_6_
363	363.1804	363.1808	1.1	C_20_H_26_O_6_
377	377.1591	377.1600	2.4	C_20_H_24_O_7_

Compared with compound 361, the element compositions of compounds 363 and 377 indicate the existence of dihydro- and hydroxyl- specific sites for these two SL components. Earlier studies have identified 5DS and 7-hydroxyorobanchyl from root exudates in some *Asteraceae* species (Yoneyama et al., 2011). Derivatization experiments on strigol and orobanchol suggested that the three putative SLs were neither Me-strigol nor Me-orobanchol (Supplementary Table S2). Using an MS^3^ scan of [M+H-CH_3_OH]^+^ from compound 361 and 363 (Supplementary Figure S3), a ring of 5DS is shown to be substituted with a methoxyl group. Based on these data, it was inferred that they share a 5DS backbone structure, which is consistent with former results, suggesting that the compounds are 5DS derivatives (Yoneyama et al., 2011).

**Figure 3:**
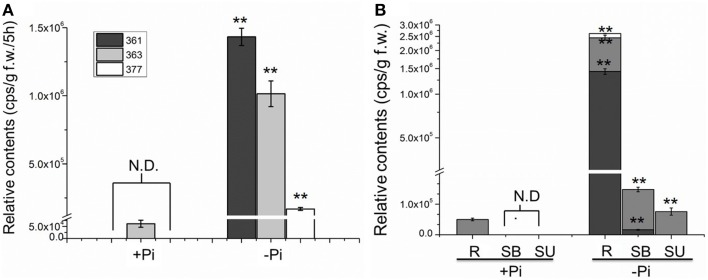
**Quantification of putative SL analogs in root exudates and whole chrysanthemum plant. (A)** SL analog levels in root exudates. N.D. not detected due to low abundance. **(B)** SL analog distribution levels in chrysanthemum. N.D. not detected due to low abundance. Means ± SE of three biological replicates are shown. Each replicates contains 60–100 plants. Comparison between Pi starvation (Pi−) and Pi sufficient (Pi+) conditions using Student's *t*-test; ^**^P < 0.01. SB, bottom parts of stems; SU, upper parts of stems; R, root.

To evaluate the relationship between Pi starvation and SL analog accumulation in chrysanthemum root exudate, the three compounds were quantified under hydroponic culture conditions of full fertilization and Pi starvation (Yoneyama et al., 2012). Exudation of all three analogs increased significantly in the Pi-depleted culture system after 2 weeks, compared with that in fully fertilized conditions. Compounds 361 (*p* = 7.53E-09) and 377 (*p* = 1.87E-05) were rarely detected in root exudates under full nutrient cultivation conditions (Figure 3A). The finding that Pi starvation induces SL analog accumulation in root exudates of chrysanthemum is consistent with earlier data obtained using different plant species (López-Ráez et al., 2008; Umehara et al., 2010; Yoneyama et al., 2012). In combination with the results obtained from a variety of plants, these Pi induction experiments confirmed that the three components are novel putative SLs in chrysanthemum.

Subsequently the distribution of endogenous SL analogs in whole plants under fully fertilized and Pi-starved conditions were examined. Chrysanthemum plantlets were separated into three parts: root (R), basal stem (SB) and upper stem (SU) (Figures 1B, 3B). Each part was fragmented, and compounds 361, 363, and 377 were quantified. These results confirmed that Pi starvation induces increased endogenous SL analog levels in all parts of the plant. Compound 377 showed low abundance *in vivo* under both culture conditions, and was only detected in the root after Pi starvation (*p* = 1.19E-05). Compound 361 was abundant in the root after Pi starvation (*p* = 2.59E-06) whereas compound 363 was relatively abundant in the stem (Figure 3B). Based on quantification of the sum of all three compounds, the highest accumulation of the analogs was in the roots. In basal stem, total accumulation was relatively higher than that in the upper stem (Figure 3B). Accordingly, it was concluded that Pi starvation reduces shoot branching in chrysanthemum and induces accumulation of SL analogs in root exudates and *in vivo*. Earlier research on rice found a negative correlation between SL levels and bud outgrowth (Umehara et al., 2010). However, in this case, the distribution of the SL level (total) was not significantly different between the upper stem and basal stem (*p* = 0.34), and was not correlated with bud length distribution along the main stem (Figures 2B, 3B). This result supports that SLs are mainly biosynthesized in roots. Incorporated knowledge from other species, SLs are transported from the basal part to the upper part, which may present a systemic means of regulation of axillary bud outgrowth in chrysanthemum.

### Isolation of the biosynthesis gene, *DgCCD7*, from chrysanthemum

Several orthologs in chrysanthemum have been identified to date, including a biosynthesis gene *DgCCD8, DgMAX2* that participates in signaling, and *DgBRC1* which is a proposed target of the SL pathway. Here, another SL biosynthesis member, *DgCCD7*, has been characterized, with the aim of using the data in conjunction with prior findings to clarify the pathway response in Pi starvation.

**Figure 4:**
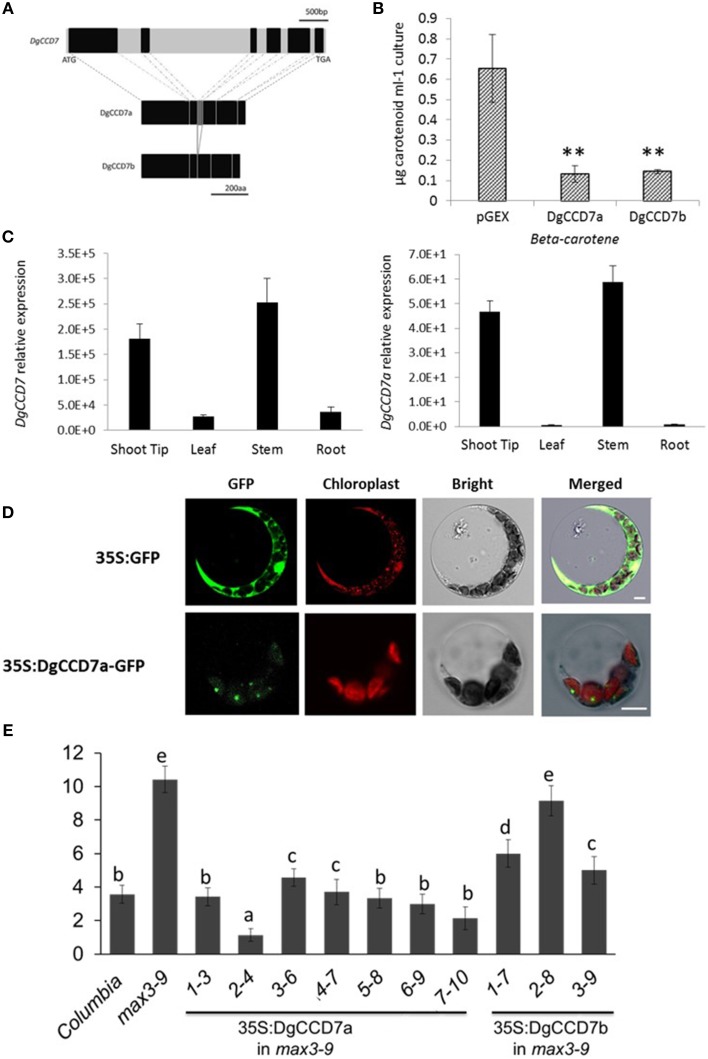
**Characterization of the SL biosynthesis gene, ***DgCCD7.*** (A)** Structures of the *DgCCD7* genes. Genomic sequence with black blocks representing exons, and introns, 5′UTR and 3′UTR in light gray. The predicted protein products of two segments isolated from cDNA are shown below, designated *DgCCD7a* and *DgCCD7b*. Scale bars above and beneath indicate the nucleic acid and amino acid length, respectively. **(B)** β-Carotene accumulation in *E.coli* cells expressing DgCCD7. ß-Carotene analysis was performed as described previously (Schwartz et al., 2004). Data are presented as means ± SE of five biological replicates, with at least 8 samples per replicate. Comparisons between pGEX and DgCCD7a/b were using Student's *t*-test; ^**^P < 0.01. **(C)** Relative expression analysis of total *DgCCD7*and *DgCCD7a* in chrysanthemum. Data obtained from quantitative real-time reverse transcription-PCR are presented as means ± SE of at least three biological replicates, with 8–10 plants per replicate. **(D)** Subcellular localization of 35S: GFP (top panel) and 35S: DgCCD7-GFP (bottom panel) in *Arabidopsis* protoplast cells. At least 20 protoplasts were examined. Bars = 10 μm. **(E)** The secondary rosette branch number of WT, *max3-9* and independent homozygous lines transformed carrying 35S::DgCCD7a and 35S::DgCCD7b in *max3-9* background. The mean number of rosette branches with a length of at least 5 mm is shown. Data represent mean numbers of branches ± SE (*n* = 16–20). The different letters denote significant differences at *P* < 0.05 in mean values as determined using Duncan's test. Values with the same lowercase letter are not significantly different from one another.

To isolate the *DgCCD7* gene, the homology cloning approach was applied. Two cDNA clones of *DgCCD7*, designated *DgCCD7a* (KT004449) and *DgCCD7b* (KT004450), were obtained. The first was 1860 bp encoding a 620-residue protein, and the second was 1728 bp encoding a 576-residue protein. Interestingly, the 44 missing amino acids of DgCCD7b, compared with DgCCD7a, encoded the third exon (Figure 4A). Phylogenetic analysis showed both DgCCD7 proteins were most closely related to putative AaCCD7 from *Artemisia annua*, with 96% similarity (Supplementary Table S3; Supplementary Figures S4A,B).

To ascertain whether the two DgCCD7 proteins display enzyme activity, we performed *in vitro* expression assays in *E.coli* (Schwartz et al., 2004). As shown, both DgCCD7s significantly degraded β-carotene in the β-carotene *E.coli* strain (Figure 4B), indicating that the missing sequence does not affect enzyme activity. We further analyzed the subcellular localization of the two proteins via a transient expression experiment in both *Arabidopsis* protoplasts and onion epidermis cells. In contrast to the control, which was ubiquitously detected in onion epidermal and protoplast cells, the DgCCD7a-GFP fusion protein was predominantly localized in chloroplasts (Figure 4D; Supplementary Figure S4C), while DgCCD7b localization was indistinct (data not shown).

The identity of DgCCD7 was further confirmed with a genetic complementation test. The plasmid containing the full-length coding sequence was introduced into a *max3-9* mutant. All transgenic lines of DgCCD7a complemented the *max3* phenotype (Figure 4E), while DgCCDb complemented the *max3* phenotype to a partial extent. Therefore, similar to the other orthologs, DgCCD7 proteins appear to play a conserved role in SL biosynthesis-the chloroplast-specific protein cleaves β-carotene and inhibits shoot branching. By now, part of SL pathway genes in chrysanthemum have been identified.

### Pi's negative regulation on the expression of SL biosynthesis gene *DgCCD7/8* in root of chrysanthemum

**Figure 5:**
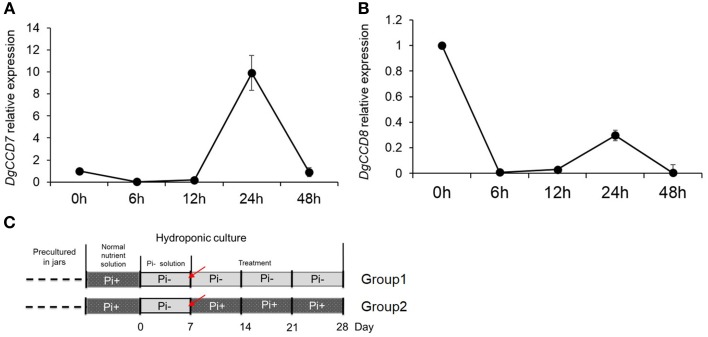
**Relative expression of SL biosynthesis genes ***DgCCD7/8*** in root after adding Pi to plants that previously starved of Pi. (A)** Relative transcript levels of *DgCCD7*. **(B)** Relative transcript levels of *DgCCD8*. **(C)** Schematic diagram for the experiment. Red arrows marked the beginning of gene transcript detection after addition of Pi. Data obtained from quantitative real-time reverse transcription-PCR are presented as means ± SE of three biological replicates, with 8–10 plants per replicate.

Previous results indicated that compared with Pi starvation group (Group1), the group of chrysanthemums in full nutrient environment (Group 2) had a lower accumulation of SLs in both root extracts and plants (Figures 1A, 3). Based on these results, the expression of SLs biosynthesis genes *DgCCD7/8* were detected in roots by transferring into full nutrient cultured condition (Group2). Group1 was used as a control (Figure 5C). The data were collected for the calculation of relative changes at each time point. The beginning of the detection time is marked with red arrow(s) (Figure 5C).

These data confirmed that transcript levels of both, *DgCCD7* and *DgCCD8*, decreased to the lowest level within 6 h after adding Pi (Figure 5). As for *DgCCD7*, after 12 h, its transcript accumulation augmented almost 9 times. But 48 h after Pi re-supply, it decreased to the original level. As for *DgCCD8*, the transcript accumulation raised a little within 24 h after Pi re-supply, but then it returned to the original level after 48 h. Nevertheless, it can be concluded that, Pi supply negatively regulated the transcript levels of SL biosynthetic genes *DgCCD7/8* in roots, but this effect lost after 6 h. In this case, Pi's negative regulation on SL biosynthesis genes might contribute to further preventing SL accumulation in root exudates in the next 2 or 3 weeks.

### SL response in node to Pi starvation and re-supply in chrysanthemum

**Figure 6:**
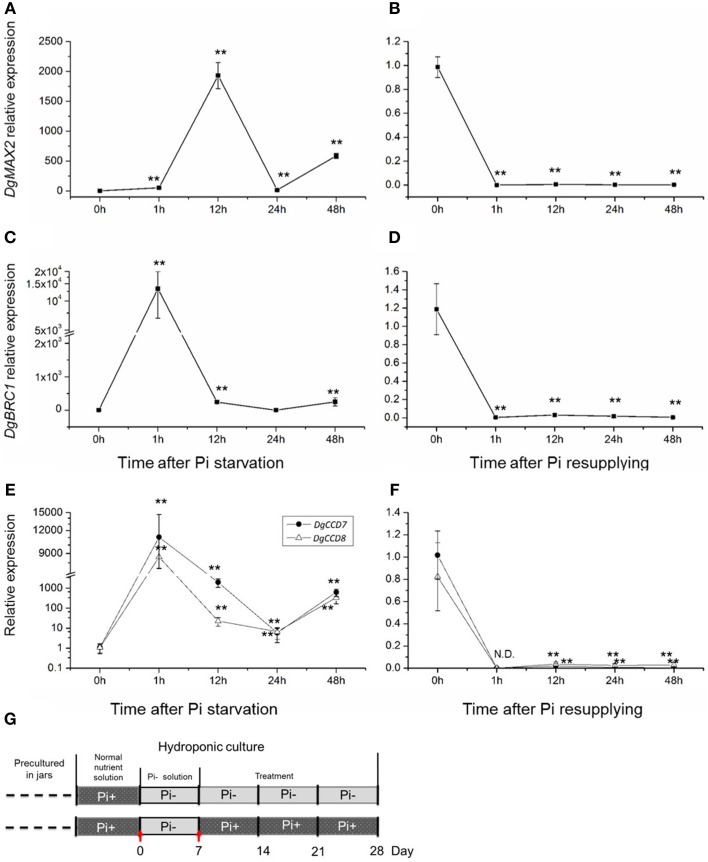
**Response of SL biosynthesis genes ***DgCCD7/8***, signaling gene, ***DgMAX2***, and the signal integrator, ***DgBRC1*** in axillary buds to Pi starvation and resupply**. **(A–D)** Transcript levels of *DgMAX2* and *DgBRC1* in nodes of chrysanthemum. **(E,F)** Transcript levels of *DgCCD7* and *DgCCD8* in nodes of chrysanthemum. **(G)** Schematic diagram for the experiment. Red arrows marked beginning of node section materials harvesting as Pi starvation at day 0 and Pi resupplying at day 7. Data obtained from quantitative real-time reverse transcription-PCR are presented as means ± SE of three biological replicates, with 9 plants per replicate. Comparisons between 0 h and other time-points using Student's *t*-test; N.D., not detected due to low abundance; ^**^P < 0.01.

To further gain an understanding of the response of the SL pathway genes to Pi in axillary buds of chrysanthemum, expression changes of the SL pathway gene in nodes (without leaves, Figure 1C) were assessed using plants that had been cultured with full nutrients which were then subjected to Pi starvation and re-supply conditions. Based on previous studies about tissue expression patterns of *DgCCD8, DgMAX2* and *DgBRC1* in chrysanthemum (Liang et al., 2010; Chen et al., 2013; Dong et al., 2013), together with *DgCCD7* (Figures 4B,C), they were also shown to be expressed relatively abundantly in the shoot tip. The beginning of the detection time is marked with a red arrow at day 0 and day 7 (Figure 6G).

It is obvious that the buds status from the upper part and basal part of the plant were different (Figures 1B, 2B). The outgrowth activity of buds located at different positions were determined (Supplementary Figure S5). All single node segments were inserted into MS medium split-plates (Liang et al., 2010; Chen et al., 2013). The elongation of each bud was measured. Bud n-12 and bud n-14 represented basal buds whereas bud n-10 and bud n-2 represented upper buds. As can be seen in 5 days, the relative elongation lengths of basal buds reached no more than 2 mm. However, upper buds can relatively elongate to more than 2.5 mm. In the first 3 days, the four positioned buds elongation was not significant. But at day 4, n-10 buds' length was significantly higher than that of the n-14 and n-12 buds. At day 5, both n-10 and n-2 buds grew more than 2.5 mm, and were significantly longer than the others (Supplementary Figure S5). The data of bud length at each day showed there were differences between upper buds and basal buds. Thus, it was considered that basal buds and upper buds were not uniform. In order to keep the materials uniform, n-1 to n-6 node sections (without leaves) were collected and mixed for the analysis (Figures 1B,C, 2B).

SLs in plant tissues were hardly detected in this case as the chrysanthemum plant subjected to Pi starvation was for less than a week. However, the gene expression pattern in buds has been largely affected by changing the culture conditions. The transcription of the SL signaling gene *DgMAX2* and bud-specific transcription factor *DgBRC1* were analyzed. They were stimulated by Pi starvation and suppressed upon resupply of Pi (Figures 6A–D). The *DgMAX2* transcription accumulated significantly 12 h after Pi depletion and decreased within 1 h of re-supply of Pi. However, the *DgBRC1* expression response to Pi starvation was evident within 1 h, considerably earlier than *DgMAX2*. *BRC1* is thought to be an integrator of branching signals and the environment (Kebrom et al., 2006; Aguilar-Martinez et al., 2007; Minakuchi et al., 2010; Braun et al., 2012; Dun et al., 2012). In chrysanthemum, *DgBRC1* was mainly expressed in dormant axillary buds and its high expression indicates the suppression response of bud outgrowth (Chen et al., 2013). Thus, in this case *DgBRC1* was able to respond to inhibition signals other than SLs as facing Pi starvation. Combined with previous results, SL shall participate in systemic regulation in response to Pi starvation.

One interesting result was the local response of the SL biosynthesis genes in the nodes themselves (Figures 6E,F). Pi starvation induced *DgCCD7* and *DgCCD8* expression with an approximately 10,000-fold increase within 1 h. This increase decreased at 24 h but increased again at 48 h. Obviously both genes kept a significantly high expression level after Pi starvation. On the contrary, re-supply of Pi led to immediate suppression of *DgCCD7/8* gene expression. Whether this induction was caused by direct Pi depletion in cultural media or was an indirect effect is still unknown. But this induction indicated that the SL pathway in the node was a local response to nutrient alterations in chrysanthemum.

### SL pathway response to Pi starvation during its delaying effect of buds outgrowth under apical auxin

**Figure 7:**
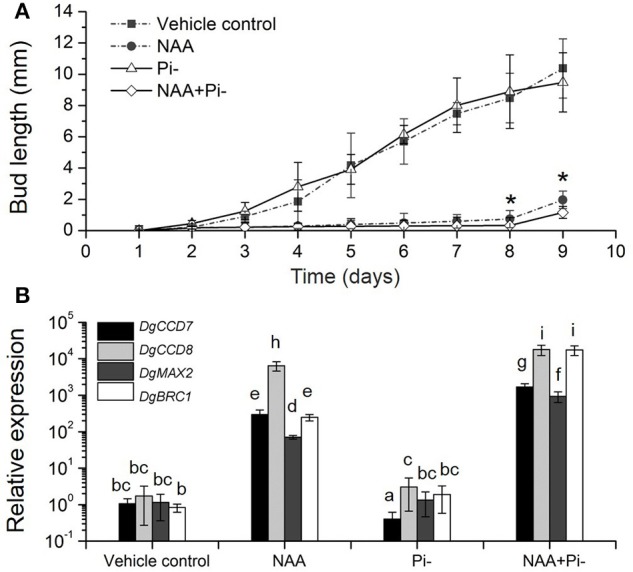
**Effects of Pi starvation and auxin (NAA) on bud outgrowth and SL pathway genes transcription in modified node stem assay. (A)** The lengths of buds were measured every 1 day. The apical block contained either 5 μM NAA or no NAA (with an equal volume of ethanol carrier), and the basal block contained the same nutrient media as the apical block, but with or without Pi. Data represent means ± SE (*n* = 20–25). Comparisons between NAA and NAA+Pi- at day 8 and day 9 using Student's *t*-test; ^*^P < 0.05. **(B)** Transcript patterns of SL pathway genes, *DgCCD7, DgCCD8, DgMAX2*, and *DgBRC1*, in modified node segments after NAA and Pi starvation treatments. Segments were treated 4 h before collection. Data obtained from quantitative real-time reverse transcription-PCR are presented as means ± SE of three biological replicates, with 8–10 plants per replicate. Letters indicate significant differences between them at a = 0.05.

We then tested the relationship among Pi starvation, auxin and axillary bud growth, to gain the overall regulation of SLs-auxin in response to Pi starvation. In whole plants, long-term Pi starvation relocated auxin to the main stems and caused the increase of SLs in chrysanthemum. Meanwhile, Pi starvation can immediate induce *DgBRC1* expression. It has been reported that transcripts of *DgBRC1* could respond to apical auxin supply and polar auxin transport (Chen et al., 2013). The modified node section assay (see Materials and Methods) was then applied. As two buds on the detected section were in same development status, we only focused on the top node (Supplementary Figure S1). According to the growth pattern, Pi absence alone, similar to GR24 alone (Liang et al., 2010), had no effect on bud elongation. NAA was able to inhibit the outgrowth of the buds with or without Pi (Figure 7A). Nevertheless, compared to NAA alone, the delay can be seen in Pi absence under apical NAA dominance after 7 days of treatment. Using ANOVA, at day 8 and day 9, treatment of NAA+Pi- significantly delayed the outgrowth of buds compared with NAA treatment alone.

To ascertain the local response of SL pathway in this process, gene transcript levels were quantified. The nodes (without leaves) were collected after 4 h of treatment. *DgCCD7* and *DgCCD8* were up-regulated significantly in the presence of apical NAA. This is in agreement with results reported previously (Liang et al., 2010). Under Pi starvation alone, *DgCCD7* was suppressed significantly, whereas *DgCCD8* was slightly up-regulated. When apical NAA was added under Pi starvation, transcription of SL biosynthesis genes was significantly increased by 2~4 orders of magnitude, compared to that observed with Pi starvation alone. Thus, NAA was able to sufficiently up-regulate the *DgCCD7* and *DgCCD8* genes transcription in the nodes. When under apical NAA plus Pi starvation conditions, induction of *DgCCD7* and *DgCCD8* in the nodes was significantly stronger than that by solo apical NAA treatment (Figure 7B). Meanwhile, *DgMAX2* and *DgBRC1* presented a similar pattern as SL biosynthesis genes. Pi starvation alone did not affect *DgMAX2* and *DgBRC1* transcription levels in this case but apical NAA can significantly enhance the induction effect. As treated with apical NAA coupled with Pi starvation, transcripts of *DgMAX2* and *DgBRC1* was strongly accumulated at a higher level than under solo NAA treatment (Figure 7B). Our data indicate that apically applied NAA is sufficient to inhibit bud outgrowth. Pi absence should facilitate a delaying effect on outgrowth of axillary buds accompanied by an auxin source. Without apical auxin, solo Pi absence played a limited role in controlling bud outgrowth; this process can be related to the node location SL pathway response. In chrysanthemum, Pi absence can amplify the inhibition effect of apical NAA accompanied induction of SL pathway genes and *DgBRC1*.

## Discussion

### Conservation of SL pathways

Conservation of SL, an essential regulator in plants, has been confirmed in a variety of plant species, based on similar components in SL pathways across different species and common backbone structures shared by functional SLs and their analogs. Extensive studies on plant models, such as Arabidopsis, rice and peas, have paved the way for greater understanding in this area.

The results from the current study showed that at least two *DgCCD7* cDNAs exist in chrysanthemum. In all CCDs, exon/intron organization is conserved and the number of exons in CCD7 ranges from 6 to 9 in dicotyledon species (Wang et al., 2011). As we only isolated one clone from genomic DNA, the reason for the missing exon in *DgCCD7b* may due to alternative splicing (Wang et al., 2011). With functional complementation of *max3-9* mutants, subcellular localization in the plastid stroma and β-carotene cleavage of *DgCCD7a* supported a conserved function. The experiments reported here showed that the missing exon leads to less complementation of *max3-9* mutants, similar to subcellular localization, but does not influence the ability to cleave β-carotene. For chrysanthemum is hexaploid, whether there are more members of CCD7s in chrysanthemum is not known but there are at least two alternatives so far.

CCD7 and CCD8 function in SL biosynthesis. As to the expression pattern, similar patterns of *DgCCD8* responses to Pi starvation and NAA/Pi- were also observed in *DgCCD7*, in both whole plants or in a spilt-plate assay. They both could be induced by Pi starvation in roots and axillary buds, and in the spilt-plate assay, they can be induced by apical NAA as well. This induction can be amplified by depleting Pi. But *DgCCD7* was suppressed significantly by Pi starvation alone while *DgCCD8* was up-regulated. This indicated that *DgCCD7* and *DgCCD8* might share a common feature in their *cis*-elements.

In this study, three putative SLs were also obtained which possess common basic 5DS backbone structure properties. Based on all the mass spectrometry data acquired from chrysanthemum, we suggest these novel compounds belong to the SL family. The activities of these putative SLs can be evaluated precisely with germination assay in our future work, however, in view of their strong induction upon Pi starvation after 3 weeks, these compounds are currently suggested to comprise the bulk of SL in chrysanthemum. Normal production of SLs is poorly detected. Nevertheless, a feasible method for quantification SLs analogs in plants using UPLC-ESI-Qtrap-MS screening was provided.

### Pi regulation of shoot branching in chrysanthemum correlated with the SL pathway

Branching architecture in the chrysanthemum was evidently different from that in Arabidopsis, rice and pea. Regardless of the culture conditions (with or without Pi), the branching habits of the chrysanthemum were distinct between the upper shoots and basal shoots (Figures 1B, 2A,B). Apical dominance in plants leads to a typical architecture where axillary bud length is associated with distance to the top bud. Previous studies have indicated that both developmental status and distance from the shoot apex influence activation of axillary buds in chrysanthemum (Thimann and Skoog, 1933; McSteen and Leyser, 2005; Chen et al., 2013). Clearly the axillary buds located below node n-12 showed relatively lower activity compared with that of the upper buds (from node n-1 to n-11) (Supplementary Figure S5). As far as we know, these three buds were from propagated cuts, which might cause a difference of branching habits between the upper shoots and the basal shoots in chrysanthemum. These experiments have revealed that axillary buds formed in the n-1 to n-11 position were uniform and may be dominated by the shoot apex (Figure 1B). Nevertheless, under long-term Pi starvation, axillary buds of chrysanthemum in whole plants were strongly inhibited, a result which is consistent with earlier findings in other species (Umehara et al., 2010; Kohlen et al., 2011; Puig et al., 2012).

Meanwhile, SLs in chrysanthemum were highly induced under Pi starvation for 28 days, both in root exudates and plantlets. Their gradient distribution indicated that SLs were secreted from roots and accumulated more in the roots than in the shoots. SL accumulation in the shoots might be because of their transportation from roots or a small amount of local biosynthesis. At the beginning of Pi starvation, *DgCCD7/8* expression was induced in the root. This transcription induction indicated further accumulation of SLs is likely with a continued absence of Pi afterwards. These results substantiate the common knowledge that SL biosynthesis is mainly found in the root and acropetally transported into shoots, and increased SL levels can be induced by Pi starvation. Furthermore, auxin relocation detected after a long period of Pi starvation suggested that auxin might participate in Pi response and as well as SLs.

In the Pi starvation and re-supply test, stimulation of *DgBRC1* by Pi absence was rapid. As *DgBRC1* is an inhibition signal integrator in buds, this finding suggested that Pi starvation triggered signals that facilitate the bud inhibition in chrysanthemum (Chen et al., 2013). The response of *DgMAX2* at axillary buds to Pi stimulation indicated Pi absence triggered the response of SL pathway as well. The local SL biosynthesis also occurred in response to Pi starvation. So it is difficult to know if local SLs contributed to bud inhibition other than systemic SL regulation in absence of Pi. These results demonstrate that in chrysanthemum, genes in the SL pathway in nodes (without leaves) respond to Pi starvation and re-supply. The relationship between *DgBRC1* and SL pathway was unknown in this case.

In this modified node segment assay, provision of an apical auxin source strongly induced the local SL biosynthesis and the signaling effect under Pi starvation. Because of multiple signals involving nutrient systemic regulation, the inhibition effect of Pi starvation is similar to that of *GR24* but it is far too weak. By comparing bud growth under different treatment (Figure 7A), Pi starvation was shown to be able to delay buds elongation only by apically applying auxin at the same time. Based on these results, node location of the SL pathway response to Pi starvation occurs at a very early stage and may contribute to a delaying effect on the elongation of the chrysanthemum buds. As the Pi absence continued, acropetally transported SLs accumulate and enhance the inhibition of the outgrowth of the axillary buds over a long period of time. However, both regulation strategies are in need of apical auxin and are coupled with alteration of the auxin status. Recent research provided evidence that Pi status is correlated with SL exudation (Yoneyama et al., 2012, 2013; Sun et al., 2014). It is supposed that Pi relocation in plants caused by Pi absence might be the reason for the triggering of the SL gene response in nodes in chrysanthemum.

The hormone network is only a small part of the overall processes mediating environmental control of shoot branching. Sugar has recently been shown to induce shoot branching and to also participate in stress responses as well (Mason et al., 2014; Van Den Ende, 2014; Barbier et al., 2015). Therefore, the fast changing of these signals could participate in activating local biosynthesis and signaling of SLs. A recent study also proposed that shoot-derived signals but not auxin are involved in the systemic regulation of SL production in roots (Yoneyama et al., 2015). It seems that signals exchanged between shoot and root response to nutrient deficiency plays an essential role in shoot branching and root architecture development. Thus, the network of mechanisms underlying the regulation of phosphorus, sugar and other nutrients that act specifically in shoot branching control require elucidation.

## Author contributions

Proofread the manuscript: LZ, NM, and CY. Conceived and designed the experiments: LX, LZ, CY, and NM. Performed the experiments: LX, CW, XC, SF, JN, CQY, and JC. Analyzed the data: LX, SF, LZ and CY. Contributed reagents/materials/analysis tools: LX, XC, CW, and CY. Wrote the paper: LX.

### Conflict of interest statement

The authors declare that the research was conducted in the absence of any commercial or financial relationships that could be construed as a potential conflict of interest.

## Abbreviations

Pi: phosphorus
SL(s): Strigolactone (s)
UPLC-MS: ultra-performance liquid chromatography coupled with mass spectrometry
LC-MS/MS: High performance liquid chromatography/tandem mass spectrometry
CCD7: CAROTENOID CLEAVAGE DIOXYGENASE 7
CCD8: CAROTENOID CLEAVAGE DIOXYGENASE 8
PIN1: PIN-FORMED1
PGRs: plant growth regulators
IAA: Indole-acetic acid.

## References

[B1] Aguilar-Martìnez J. A. Poza-Carrión C. Cubas P. (2007). Arabidopsis BRANCHED1 acts as an integrator of branching signals within axillary buds. Plant Cell 19, 458–472. 10.1105/tpc.106.048934 PMC1867329 17307924

[B2] Alder A. Jamil M. Marzorati M. Bruno M. Vermathen M. Bigler P.. (2012). The path from beta-carotene to carlactone, a strigolactone-like plant hormone. Science 335, 1348–1351. 10.1126/science.1218094 22422982

[B3] Arite T. Iwata H. Ohshima K. Maekawa M. Nakajima M. Kojima M.. (2007). DWARF10, an RMS1/MAX4/DAD1 ortholog, controls lateral bud outgrowth in rice. Plant J. 51, 1019–1029. 10.1111/j.1365-313X.2007.03210.x 17655651

[B4] Balla J. Kalousek P. Reinöhl V. Friml J. Prochazka S. (2011). Competitive canalization of PIN-dependent auxin flow from axillary buds controls pea bud outgrowth. Plant J. 65, 571–577. 10.1111/j.1365-313X.2010.04443.x 21219506

[B5] Barbier F. Peron T. Lecerf M. Perez-Garcia M. D. Barriere Q. Rolcik J.. (2015). Sucrose is an early modulator of the key hormonal mechanisms controlling bud outgrowth in Rosa hybrida. J. Exp. Bot. 66, 2569–2582. 10.1093/jxb/erv047 25873679 PMC4986866

[B6] Booker J. Sieberer T. Wright W. Williamson L. Willett B. Stirnberg P.. (2005). MAX1 encodes a cytochrome P450 family member that acts downstream of MAX3/4 to produce a carotenoid-derived branch-inhibiting hormone. Dev. Cell 8, 443–449. 10.1016/j.devcel.2005.01.009 15737939

[B7] Braun N. De Saint Germain A. Pillot J. P. Boutet-Mercey S. Dalmais M. Antoniadi I.. (2012). The pea TCP transcription factor PsBRC1 acts downstream of Strigolactones to control shoot branching. Plant Physiol. 158, 225–238. 10.1104/pp.111.182725 22045922 PMC3252107

[B8] Brewer P. B. Dun E. A. Ferguson B. J. Rameau C. Beveridge C. A. (2009). Strigolactone acts downstream of auxin to regulate bud outgrowth in pea and Arabidopsis. Plant Physiol. 150, 482–493. 10.1104/pp.108.134783 19321710 PMC2675716

[B9] Brewer P. B. Dun E. A. Gui R. Mason M. G. Beveridge C. A. (2015). Strigolactone inhibition of branching independent of polar auxin transport. Plant Physiol. 168, 1820–1829. 10.1104/pp.15.00014 26111543 PMC4528729

[B10] Brewer P. B. Koltai H. Beveridge C. A. (2013). Diverse roles of strigolactones in plant development. Mol. Plant 6, 18–28. 10.1093/mp/sss130 23155045

[B11] Chatfield S. P. Stirnberg P. Forde B. G. Leyser O. (2000). The hormonal regulation of axillary bud growth in Arabidopsis. Plant J. 24, 159–169. 10.1046/j.1365-313x.2000.00862.x 11069691

[B12] Chen X. Zhou X. Xi L. Li J. Zhao R. Ma N.. (2013). Roles of DgBRC1 in regulation of lateral branching in chrysanthemum (Dendranthema xgrandiflora cv. Jinba). PLoS ONE 8:e61717. 10.1371/journal.pone.0061717 23613914 PMC3629106

[B13] Clough S. J. Bent A. F. (1998). Floral dip: a simplified method for Agrobacterium-mediated transformation of Arabidopsis thaliana. Plant J. 16, 735–743. 10.1046/j.1365-313x.1998.00343.x 10069079

[B14] Cook C. E. Whichard L. P. Turner B. Wall M. E. Egley G. H. (1966). Germination of witchweed (Striga lutea Lour.): isolation and properties of a potent stimulant. Science 154, 1189–1190. 10.1126/science.154.3753.1189 17780042

[B15] Crawford S. Shinohara N. Sieberer T. Williamson L. George G. Hepworth J.. (2010). Strigolactones enhance competition between shoot branches by dampening auxin transport. Development 137, 2905–2913. 10.1242/dev.051987 20667910

[B16] Cunningham F. X. Jr. Sun Z. Chamovitz D. Hirschberg J. Gantt E. (1994). Molecular structure and enzymatic function of lycopene cyclase from the cyanobacterium Synechococcus sp strain PCC7942. Plant Cell 6, 1107–1121. 10.1105/tpc.6.8.1107 7919981 PMC160505

[B17] de Jong M. George G. Ongaro V. Williamson L. Willetts B. Ljung K.. (2014). Auxin and strigolactone signaling are required for modulation of Arabidopsis shoot branching by nitrogen supply. Plant Physiol. 166, 384–395. 10.1104/pp.114.242388 25059707 PMC4149722

[B18] Deruiter H. A. (1996). Development of chrysanthemum cuttings: the influence of age and position of the axillary buds. Ann. Bot. 77, 99–104. 10.1006/anbo.1996.0012

[B19] Domagalska M. A. Leyser O. (2011). Signal integration in the control of shoot branching. Nat. Rev. Mol. Cell Biol. 12, 211–221. 10.1038/nrm3088 21427763

[B20] Dong L. Ishak A. Yu J. Zhao R. Zhao L. (2013). Identification and Functional Analysis of Three MAX2 Orthologs in Chrysanthemum. J. Integr. Plant Biol. 55, 434–442. 10.1111/jipb.12028 23302095

[B21] Drummond R. S. Janssen B. J. Luo Z. Oplaat C. Ledger S. E. Wohlers M. W.. (2015). Environmental control of branching in petunia. Plant Physiol. 168, 735–751. 10.1104/pp.15.00486 25911529 PMC4453797

[B22] Dun E. A. de Saint Germain A. Rameau C. Beveridge C. A. (2012). Antagonistic action of strigolactone and cytokinin in bud outgrowth control. Plant Physiol. 158, 487–498. 10.1104/pp.111.186783 22042819 PMC3252097

[B23] Dun E. A. De Saint Germain A. Rameau C. Beveridge C. A. (2013). Dynamics of strigolactone function and shoot branching responses in Pisum sativum. Mol. Plant 6, 128–140. 10.1093/mp/sss131 23220942

[B24] Foo E. Bullier E. Goussot M. Foucher F. Rameau C. Beveridge C. A. (2005). The branching gene RAMOSUS1 mediates interactions among two novel signals and auxin in pea. Plant Cell 17, 464–474. 10.1105/tpc.104.026716 15659639 PMC548819

[B25] Gaiji N. Cardinale F. Prandi C. Bonfante P. Ranghino G. (2012). The computational-based structure of Dwarf14 provides evidence for its role as potential strigolactone receptor in plants. BMC Res. Notes 5:307. 10.1186/1756-0500-5-307 22713366 PMC3436726

[B26] Gomez-Roldan V. Fermas S. Brewer P. B. Puech-Pagés V. Dun E. A. Pillot J. P.. (2008). Strigolactone inhibition of shoot branching. Nature 455, 189–194. 10.1038/nature07271 18690209

[B27] Greb T. Clarenz O. Schafer E. Muller D. Herrero R. Schmitz G.. (2003). Molecular analysis of the LATERAL SUPPRESSOR gene in Arabidopsis reveals a conserved control mechanism for axillary meristem formation. Genes Dev. 17, 1175–1187. 10.1101/gad.260703 12730136 PMC196050

[B28] Guan J. C. Koch K. E. Suzuki M. Wu S. Latshaw S. Petruff T.. (2012). Diverse Roles of Strigolactone Signaling in Maize Architecture and the Uncoupling of a Branching-Specific Subnetwork. Plant Physiol. 160, 1303–1317. 10.1104/pp.112.204503 22961131 PMC3490586

[B29] Hamiaux C. Drummond R. S. Janssen B. J. Ledger S. E. Cooney J. M. Newcomb R. D.. (2012). DAD2 is an alpha/beta hydrolase likely to be involved in the perception of the plant branching hormone, strigolactone. Curr. Biol. 22, 2032–2036. 10.1016/j.cub.2012.08.007 22959345

[B30] Hayward A. Stirnberg P. Beveridge C. Leyser O. (2009). Interactions between auxin and strigolactone in shoot branching control. Plant Physiol. 151, 400–412. 10.1104/pp.109.137646 19641034 PMC2735998

[B31] Kapulnik Y. Delaux P. M. Resnick N. Mayzlish-Gati E. Wininger S. Bhattacharya C.. (2011). Strigolactones affect lateral root formation and root-hair elongation in Arabidopsis. Planta 233, 209–216. 10.1007/s00425-010-1310-y 21080198

[B32] Kebrom T. H. Burson B. L. Finlayson S. A. (2006). Phytochrome B represses Teosinte Branched1 expression and induces sorghum axillary bud outgrowth in response to light signals. Plant Physiol. 140, 1109–1117. 10.1104/pp.105.074856 16443694 PMC1400571

[B33] Kohlen W. Charnikhova T. Liu Q. Bours R. Domagalska M. A. Beguerie S.. (2011). Strigolactones are transported through the xylem and play a key role in shoot architectural response to phosphate deficiency in nonarbuscular mycorrhizal host Arabidopsis. Plant Physiol. 155, 974–987. 10.1104/pp.110.164640 21119045 PMC3032481

[B34] Koltai H. (2013). Strigolactones activate different hormonal pathways for regulation of root development in response to phosphate growth conditions. Ann. Bot. 112, 409–415. 10.1093/aob/mcs216 23059852 PMC3698373

[B35] Koyama T. Furutani M. Tasaka M. Ohme-Takagi M. (2007). TCP transcription factors control the morphology of shoot lateral organs via negative regulation of the expression of boundary-specific genes in Arabidopsis. Plant Cell 19, 473–484. 10.1105/tpc.106.044792 17307931 PMC1867346

[B36] Laishram N. Dhiman S. R. Gupta Y. C. Bhardwaj S. K. Singh A. (2013). Microbial dynamics and physico-chemical properties of soil in the rhizosphere of chrysanthemum (Dendranthema grandiflora) as influenced by integrated nutrient management. Indian J. Agri. Sci. 83, 447–455.

[B37] Liang J. Zhao L. Challis R. Leyser O. (2010). Strigolactone regulation of shoot branching in chrysanthemum (Dendranthema grandiflorum). J. Exp. Bot. 61, 3069–3078. 10.1093/jxb/erq133 20478970 PMC2892150

[B38] Lin H. Wang R. Qian Q. Yan M. Meng X. Fu Z.. (2009). DWARF27, an iron-containing protein required for the biosynthesis of strigolactones, regulates rice tiller bud outgrowth. Plant Cell 21, 1512–1525. 10.1105/tpc.109.065987 19470589 PMC2700539

[B39] Livak K. J. Schmittgen T. D. (2001). Analysis of relative gene expression data using real-time quantitative PCR and the 2−ΔΔCT method. Methods 25, 402–408. 10.1006/meth.2001.1262 11846609

[B40] López-Ráez J. A. Charnikhova T. Gomez-Roldan V. Matusova R. Kohlen W. De Vos R.. (2008). Tomato strigolactones are derived from carotenoids and their biosynthesis is promoted by phosphate starvation. New Phytol. 178, 863–874. 10.1111/j.1469-8137.2008.02406.x 18346111

[B41] Mason M. G. Ross J. J. Babst B. A. Wienclaw B. N. Beveridge C. A. (2014). Sugar demand, not auxin, is the initial regulator of apical dominance. Proc. Natl. Acad. Sci. U.S.A. 111, 6092–6097. 10.1073/pnas.1322045111 24711430 PMC4000805

[B42] McSteen P. Leyser O. (2005). Shoot branching. Annu. Rev. Plant Biol. 56, 353–374. 10.1146/annurev.arplant.56.032604.144122 15862100

[B43] Minakuchi K. Kameoka H. Yasuno N. Umehara M. Luo L. Kobayashi K.. (2010). FINE CULM1 (FC1) works downstream of strigolactones to inhibit the outgrowth of axillary buds in rice. Plant Cell Physiol. 51, 1127–1135. 10.1093/pcp/pcq083 20547591 PMC2900823

[B44] Murashige T. Skoog F. (1962). A revised medium for rapid growth and bio assays with tobacco tissue cultures. Physiol. Plant. 15, 473–497. 10.1111/j.1399-3054.1962.tb08052.x

[B45] Ongaro V. Leyser O. (2008). Hormonal control of shoot branching. J. Exp. Bot. 59, 67–74. 10.1093/jxb/erm134 17728300

[B46] Prusinkiewicz P. Crawford S. Smith R. S. Ljung K. Bennett T. Ongaro V.. (2009). Control of bud activation by an auxin transport switch. Proc. Natl. Acad. Sci. U.S.A. 106, 17431–17436. 10.1073/pnas.0906696106 19805140 PMC2751654

[B47] Puig J. Pauluzzi G. Guiderdoni E. Gantet P. (2012). Regulation of shoot and root development through mutual signaling. Mol. Plant 5, 974–983. 10.1093/mp/sss047 22628542

[B48] Rubio-Moraga A. Ahrazem O. Pérez-Clemente R. M. Gómez-Cadenas A. Yoneyama K. López-Ráez J. A.. (2014). Apical dominance in saffron and the involvement of the branching enzymes CCD7 and CCD8 in the control of bud sprouting. BMC Plant Biol. 14:171. 10.1186/1471-2229-14-171 24947472 PMC4077219

[B49] Ruyter-Spira C. Kohlen W. Charnikhova T. van Zeijl A. van Bezouwen L. de Ruijter N.. (2011). Physiological effects of the synthetic strigolactone analog GR24 on root system architecture in Arabidopsis: another belowground role for strigolactones? Plant Physiol. 155, 721–734. 10.1104/pp.110.166645 21119044 PMC3032462

[B50] Sato D. Awad A. A. Chae S. H. Yokota T. Sugimoto Y. Takeuchi Y.. (2003). Analysis of strigolactones, germination stimulants for striga and orobanche, by high-performance liquid chromatography/tandem mass spectrometry. J. Agric. Food Chem. 51, 1162–1168. 10.1021/jf025997z 12590450

[B51] Schwartz S. H. Qin X. Loewen M. C. (2004). The biochemical characterization of two carotenoid cleavage enzymes from Arabidopsis indicates that a carotenoid-derived compound inhibits lateral branching. J. Biol. Chem. 279, 46940–46945. 10.1074/jbc.M409004200 15342640

[B52] Shinohara N. Taylor C. Leyser O. (2013). Strigolactone can promote or inhibit shoot branching by triggering rapid depletion of the auxin efflux protein PIN1 from the plasma membrane. PLoS Biol. 11:e1001474. 10.1371/journal.pbio.1001474 23382651 PMC3558495

[B53] Stirnberg P. Furner I. J. Ottoline Leyser H. M. (2007). MAX2 participates in an SCF complex which acts locally at the node to suppress shoot branching. Plant J. 50, 80–94. 10.1111/j.1365-313X.2007.03032.x 17346265

[B54] Sun H. Tao J. Liu S. Huang S. Chen S. Xie X.. (2014). Strigolactones are involved in phosphate- and nitrate-deficiency-induced root development and auxin transport in rice. J. Exp. Bot. 65, 6735–6746. 10.1093/jxb/eru029 24596173 PMC4246174

[B55] Thimann K. V. Skoog F. (1933). Studies on the growth hormone of plants: III. the inhibiting action of the growth substance on bud development. Proc. Natl. Acad. Sci. U.S.A. 19, 714–716. 10.1073/pnas.19.7.714 16577553 PMC1086139

[B56] Tocquin P. Corbesier L. Havelange A. Pieltain A. Kurtem E. Bernier G.. (2003). A novel high efficiency, low maintenance, hydroponic system for synchronous growth and flowering of Arabidopsis thaliana. BMC Plant Biol. 3:2. 10.1186/1471-2229-3-2 12556248 PMC150571

[B57] Umehara M. Hanada A. Magome H. Takeda-Kamiya N. Yamaguchi S. (2010). Contribution of strigolactones to the inhibition of tiller bud outgrowth under phosphate deficiency in rice. Plant Cell Physiol. 51, 1118–1126. 10.1093/pcp/pcq084 20542891 PMC2900824

[B58] Umehara M. Hanada A. Yoshida S. Akiyama K. Arite T. Takeda-Kamiya N.. (2008). Inhibition of shoot branching by new terpenoid plant hormones. Nature 455, 195–200. 10.1038/nature07272 18690207

[B59] Van Den Ende W. (2014). Sugars take a central position in plant growth, development and, stress responses. A focus on apical dominance. Front. Plant Sci. 5:313. 10.3389/fpls.2014.00313 25071796 PMC4074781

[B60] Varagona M. J. Schmidt R. J. Raikhel N. V. (1992). Nuclear localization signal(s) required for nuclear targeting of the maize regulatory protein Opaque-2. Plant Cell 4, 1213–1227. 10.1105/tpc.4.10.1213 1332794 PMC160209

[B61] Vogel J. T. Walter M. H. Giavalisco P. Lytovchenko A. Kohlen W. Charnikhova T.. (2010). SlCCD7 controls strigolactone biosynthesis, shoot branching and mycorrhiza-induced apocarotenoid formation in tomato. Plant J. 61, 300–311. 10.1111/j.1365-313X.2009.04056.x 19845881

[B62] Waldie T. McCulloch H. Leyser O. (2014). Strigolactones and the control of plant development: lessons from shoot branching. Plant J. 79, 607–622. 10.1111/tpj.12488 24612082

[B63] Wang R. K. Lu J. J. Xing G. N. Gai J. Y. Zhao T. J. (2011). Molecular evolution of two consecutive carotenoid cleavage dioxygenase genes in strigolactone biosynthesis in plants. Genet. Mol. Res. 10, 3664–3673. 10.4238/2011.December.2.2 22180067

[B64] Wang Y. Li B. Du M. Eneji A. E. Wang B. Duan L.. (2012). Mechanism of phytohormone involvement in feedback regulation of cotton leaf senescence induced by potassium deficiency. J. Exp. Bot. 63, 5887–5901. 10.1093/jxb/ers238 22962680 PMC3467299

[B65] Ward S. P. Salmon J. Hanley S. J. Karp A. Leyser O. (2013). Using Arabidopsis to study shoot branching in biomass willow. Plant Physiol. 162, 800–811. 10.1104/pp.113.218461 23610219 PMC3668071

[B66] Waters M. T. Brewer P. B. Bussell J. D. Smith S. M. Beveridge C. A. (2012). The Arabidopsis ortholog of rice DWARF27 acts upstream of MAX1 in the control of plant development by strigolactones. Plant Physiol. 159, 1073–1085. 10.1104/pp.112.196253 22623516 PMC3387695

[B67] Weiler E. W. Jourdan P. S. Conrad W. (1981). Levels of indole-3-acetic acid in intact and decapitated coleoptiles as determined by a specific and highly sensitive solid-phase enzyme immunoassay. Planta 153, 561–571. 10.1007/BF00385542 24275876

[B68] Yokota T. Sakai H. Okuno K. Yoneyama K. Takeuchi Y. (1998). Alectrol and orobanchol, germination stimulants for Orobanche minor, from its host red clover. Phytochemistry 49, 1967–1973. 10.1016/S0031-9422(98)00419-1

[B69] Yoneyama K. Kisugi T. Xie X. Arakawa R. Ezawa T. Nomura T.. (2015). Shoot-derived signals other than auxin are involved in systemic regulation of strigolactone production in roots. Planta 241, 687–698. 10.1007/s00425-014-2208-x 25417194

[B70] Yoneyama K. Xie X. Kim H. I. Kisugi T. Nomura T. Sekimoto H.. (2012). How do nitrogen and phosphorus deficiencies affect strigolactone production and exudation? Planta 235, 1197–1207. 10.1007/s00425-011-1568-8 22183123 PMC3362704

[B71] Yoneyama K. Xie X. Kisugi T. Nomura T. (2013). Nitrogen and phosphorus fertilization negatively affects strigolactone production and exudation in sorghum. Planta 238, 885–894. 10.1007/s00425-013-1943-8 23925853

[B72] Yoneyama K. Xie X. Kisugi T. Nomura T. Sekimoto H. Yokota T. (2011). Characterization of strigolactones exuded by Asteraceae plants. Plant Growth Regul. 65, 495–504. 10.1007/s10725-011-9620-z

[B73] Yoo S. D. Cho Y. H. Sheen J. (2007). Arabidopsis mesophyll protoplasts: a versatile cell system for transient gene expression analysis. Nat. Protoc. 2, 1565–1572. 10.1038/nprot.2007.199 17585298

[B74] Zhang Y. van Dijk A. D. J. Scaffidi A. Flematti G. R. Hofmann M. Charnikhova T.. (2014). Rice cytochrome P450 MAX1 homologs catalyze distinct steps in strigolactone biosynthesis. Nat. Chem. Biol. 10, 1028. 10.1038/nchembio.1660 25344813

[B75] Zhao J. Li G. Yi G. X. Wang B. M. Deng A. X. Nan T. G.. (2006). Comparison between conventional indirect competitive enzyme-linked immunosorbent assay (icELISA) and simplified icELISA for small molecules. Anal. Chim. Acta 571, 79–85. 10.1016/j.aca.2006.04.060 17723423

[B76] Zhou F. Lin Q. Zhu L. Ren Y. Zhou K. Shabek N.. (2013). D14-SCF(D3)-dependent degradation of D53 regulates strigolactone signalling. Nature 504, 406–410. 10.1038/nature12878 24336215 PMC4096652

[B77] Zou J. Zhang S. Zhang W. Li G. Chen Z. Zhai W.. (2006). The rice HIGH-TILLERING DWARF1 encoding an ortholog of Arabidopsis MAX3 is required for negative regulation of the outgrowth of axillary buds. Plant J. 48, 687–698. 10.1111/j.1365-313X.2006.02916.x 17092317

