# Supplementary material for: Impacts of strigolactone on shoot branching under phosphate starvation in chrysanthemum (Dendranthema grandiflorum cv. Jinba)
Source: Front Plant Sci. 2015 Sep 11;6:694. doi: 10.3389/fpls.2015.00694 (PMC4566059; doi:10.3389/fpls.2015.00694)
Supplement: Supplementary file 1 [file Table1.DOCX]

***Supplementary Material***

**Impacts of Strigolactone on Shoot Branching under Phosphate Starvation in Chrysanthemum**

**Lin Xi^1^, Chao Wen^1^, Shuang Fang^2^, Xiaoli Chen^1^, Jing nie^1^, JinFang Chu^2^, Cunquan Yuan^1^, Cunyu Yan 2^a§^, Nan Ma^1§^, Liangjun Zhao ^1§*^**

**^1^** Beijing Key Laboratory of Development and Quality Control of Ornamental Crops, Department of Ornamental Horticulture and Landscape Architecture, China Agricultural University, Beijing 100193, China
**^2^** National Centre for Plant Gene Research (Beijing), Institute of Genetics and Developmental Biology, Chinese Academy of Sciences, Beijing 100101, China
**a**. Current address: MIB & School of Chemistry, the University of Manchester, 131 Princess Street, Manchester M1 7DN, UK.
**§** Both authors have contributed equally to the work *** Correspondence:** **Liangjun Zhao**, Beijing Key Laboratory of Development and Quality Control of Ornamental Crops, Department of Ornamental Horticulture and Landscape Architecture, China Agricultural University, Yuanmingyuan West Road, , Beijing 100193, China
zhaolj5073@sina.com

**Supplementary Table**

## Supplementary Tables

**Supplementary Table S1. Q-TOF MS^2^ Daughter ion information of chrysanthemum SLs analogs**

| Ion Type | Daughter Ion m/z | | | | |
| --- | --- | --- | --- | --- | --- |
|  | Component361 | Component 363 | | | Component 377 |
| [M+H-CH_3_OH]^+^ | 329.1374 | | 331.1536 | 345.1331 | |
| [M+H-H_2_O]^+^ | ／ | | 345.1700 | ／ | |
| [M+H-2H_2_O]^+^ | ／ | | 327.1611 | ／ | |
| [M+H-H_2_O-CO]^+^ | 315.1616 | | 317.1758 | ／ | |
| [M+H-CH_3_OH-CO]^+^ | 301.1472 | | 303.1626 | 317.1342 | |
| [M+H-CH_3_OH-2H_2_O]^+^ | 293.1136 | | 295.1420 | ／ | |
| [M+H-H_2_O-2CO]^+^ | ／ | | 289.1104 | ／ | |
| [M+H-CH_3_OH-H_2_O-CO]^+^ | 283.1315 | | 285.1469 | 299.1269 | |
| [M+H-CH_3_OH-2CO]^+^ | 273.0743 | | 275.0927 | ／ | |
| [M+H-CH_3_OH-H_2_O-2CO]^+^ | 255.1385 | | 257.0810 | ／ | |
| [M+H-CH_3_OH-2H_2_O-CO]^+^ | ／ | | 267.1396 | ／ | |
| [M+H-CH_3_OH-2H_2_O-2CO]^+^ | ／ | | 239.0721 | ／ | |
| [M+H-D]^+•^ | 264.1354 | | 266.1505 | ／ | |
| [M+H-D]^+^ | 265.1421 | | 267.1568 | ／ | |
| [M+H-D-H_2_O]^+^ | 247.1319 | | 249.1479 | 263.1293 | |
| [M+H-D-CH_3_OH]^+^ | 233.1171 | | 235.1328 | 249.1136 | |
| [M+H-D-CH_3_OH-H_2_O]^+•^ | 214.0988 | | 216.1115 | ／ | |
| [M+H-D-CH_3_OH-H_2_O]^+^ | 215.1060 | | 217.1217 | 231.1022 | |
| [M+H-D-CH_3_OH-CO]^+^ | 205.1216 | | ／ | ／ | |
| [D]^+^ | 97.0279 | | 97.0284 | 97.0297 | |
| [D-CO]^+^ | 69.0323 | | 69.0332 | 69.0320 | |
