# Supplementary material for: Impacts of strigolactone on shoot branching under phosphate starvation in chrysanthemum (Dendranthema grandiflorum cv. Jinba)
Source: Front Plant Sci. 2015 Sep 11;6:694. doi: 10.3389/fpls.2015.00694 (PMC4566059; doi:10.3389/fpls.2015.00694)
Supplement: Supplementary file 2 [file Table2.DOCX]

***Supplementary Material***

**Impacts of Strigolactone on Shoot Branching under Phosphate Starvation in Chrysanthemum**

**Lin Xi^1^, Chao Wen^1^, Shuang Fang^2^, Xiaoli Chen^1^, Jing nie^1^, JinFang Chu^2^, Cunquan Yuan^1^, Cunyu Yan 2^a§^, Nan Ma^1§^, Liangjun Zhao ^1§*^**

**^1^** Beijing Key Laboratory of Development and Quality Control of Ornamental Crops, Department of Ornamental Horticulture and Landscape Architecture, China Agricultural University, Beijing 100193, China
**^2^** National Centre for Plant Gene Research (Beijing), Institute of Genetics and Developmental Biology, Chinese Academy of Sciences, Beijing 100101, China
**a**. Current address: MIB & School of Chemistry, the University of Manchester, 131 Princess Street, Manchester M1 7DN, UK.
**§** Both authors have contributed equally to the work *** Correspondence:** **Liangjun Zhao**, Beijing Key Laboratory of Development and Quality Control of Ornamental Crops, Department of Ornamental Horticulture and Landscape Architecture, China Agricultural University, Yuanmingyuan West Road, , Beijing 100193, China
zhaolj5073@sina.com

**Supplementary Table**

**Supplementary Table S2. Q-TOF MS^2^ Daughter ion information of derivatives of Me-Strigol and Me-Orobanchol.**

| Ion Type | Daughter Ion m/z | | |
| --- | --- | --- | --- |
|  | Me-Strigol-1 | Me-Strigol-2 | Me-Orobanchol |
| [M+H-CH_3_OH]^+^ | 329.1382 | 329.1390 | 329.1387 |
| [M+H-H_2_O]^+^ | 343.1518 | 343.1543 | ／ |
| [M+H-H_2_O-CO]^+^ | 315.1590 | 315.1608 | ／ |
| [M+H-CH_3_OH-H_2_O]^+^ | 311.1297 | 311.1277 | 311.1275 |
| [M+H-CH_3_OH-CO]^+^ | ／ | ／ | 301.1434 |
| [M+H-CH_3_OH-2H_2_O]^+^ | 293.1201 | 293.1194 | ／ |
| [M+H-CH_3_OH-H_2_O-CO]^+^ | 283.1315 | 283.1334 | 283.1342 |
| [M+H-CH_3_OH-2CO]^+^ | ／ | ／ | 273.1468 |
| [M+H-CH_3_OH-H_2_O-2CO]^+^ | 255.1406 | 255.1355 | 255.1375 |
| [M+H-CH_3_OH-2H_2_O-CO]^+^ | 237.1276 | 237.1295 | ／ |
| [M+H-D]^+•^ | ／ | ／ | 264.1389 |
| [M+H-D]^+^ | 265.1384 | 265.1416 | 265.1441 |
| [M+H-D-H_2_O]^+^ | 245.1184 | 245.1180 | ／ |
| [M+H-D-CH_3_OH]^+•^ | ／ | ／ | 232.1096 |
| [M+H-D-CH_3_OH]^+^ | 233.1169 | 233.1187 | 233.1171 |
| [M+H-D-CH_3_OH-H_2_O]^+^ | 215.1070 | 215.1072 | 215.1069 |
| [M+H-D-CH_3_OH-CO]^+^ | ／ | ／ | 205.1228 |
| [M+H-D-CH_3_OH-H_2_O-CO]^+^ | 187.1111 | 187.1135 | ／ |
| [D]^+^ | 97.0290 | 97.0290 | 97.0290 |
| [D-CO]^+^ | 69.0353 | 69.0323 | 69.0353 |
