# Supplementary material for: Impacts of strigolactone on shoot branching under phosphate starvation in chrysanthemum (Dendranthema grandiflorum cv. Jinba)
Source: Front Plant Sci. 2015 Sep 11;6:694. doi: 10.3389/fpls.2015.00694 (PMC4566059; doi:10.3389/fpls.2015.00694)
Supplement: Supplementary file 3 [file Table3.DOCX]

***Supplementary Material***

**Impacts of Strigolactone on Shoot Branching under Phosphate Starvation in Chrysanthemum**

**Lin Xi^1^, Chao Wen^1^, Shuang Fang^2^, Xiaoli Chen^1^, Jing nie^1^, JinFang Chu^2^, Cunquan Yuan^1^, Cunyu Yan 2^a§^, Nan Ma^1§^, Liangjun Zhao ^1§*^**

**^1^** Beijing Key Laboratory of Development and Quality Control of Ornamental Crops, Department of Ornamental Horticulture and Landscape Architecture, China Agricultural University, Beijing 100193, China
**^2^** National Centre for Plant Gene Research (Beijing), Institute of Genetics and Developmental Biology, Chinese Academy of Sciences, Beijing 100101, China
**a**. Current address: MIB & School of Chemistry, the University of Manchester, 131 Princess Street, Manchester M1 7DN, UK.
**§** Both authors have contributed equally to the work *** Correspondence:** **Liangjun Zhao**, Beijing Key Laboratory of Development and Quality Control of Ornamental Crops, Department of Ornamental Horticulture and Landscape Architecture, China Agricultural University, Yuanmingyuan West Road, , Beijing 100193, China
zhaolj5073@sina.com

**Supplementary Table**

**Supplementary Table S3. Oligo Primers used.**

| ID | Sequence | Purpose |
| --- | --- | --- |
| *CCD7*M-F | TGAHAMAATDACAGCTTACTGGGAT | Fragment amplifying |
| *CCD7*M-R | GTRAADKTACTGCGWGGKTGGTGRATAT | Fragment amplifying |
| 3’RACE-AUAP | GGCCACGCGTCGACTAGTACT | 3’RACE |
| 3’RACE-AP | GGCCACGCGTCGACTAGTACT(17) | 3’RACE cDNA synthesis |
| 3’-F1 | CATCGTTGGATACCCTTGGAAAGTTCGA | 3’RACE |
| 3’-F2 | TATGCTCCTACCACGCAGTACATTCA | 3’RACE |
| 5‘RACE-AAP | GGCCACGCGTCGACTAGTACGGIIGGGIIGGGGIIG | 5’RACE |
| 5‘RACE-AUAP | GGCCACGCGTCGACTAGTACG | 5’RACE |
| 5’-R1 | CCTTCTCATCACACTCTTTA | 5’RACE cDNA synthesis |
| 5’-R2 | TGGTCTTAATGTATCTAGCC | 5’RACE |
| 5’-R3 | ACGCTCTAAGATAGCCATGACCA | 5’RACE |
| *CCD7*F-F1 | AAATGTCAATTCCTAAAACACTCG | Full-length amplifying |
| *CCD7*F-R1 | CCACAATAGAAATAGAACACTAGGCAG | Full-length amplifying |
| *CCD7*F-F2 | AGCACCATTCCTACTAAGTTATTAT | Full-length amplifying |
| *CCD7*F-R2 | ATGGTTTAGTCTACATTTTTTGAATCAGCAG | Full-length amplifying |
| *CCD7 tra*-F | CCCAAGCTT ATGCAGGCCAAAGCATTCAAT | pCAMBIA1300 construction |
| *CCD7 tra*-R | CATGGACGAGCTGTACAAGTAAGGTACCCCG | pCAMBIA1300 construction |
| *CCD7-GFP* fusion-F | CCCAAGCTTATGCAGGCCAAAGCATTCAAT | pEZS-NL construction |
| *CCD7-GFP* fusion-R | GGGCAGCTGCTGATTCAAAAATGTTGGATCCGCG | pEZS-NL construction |
| *CCD7a* RT-F | TGATGCTCCAAGAAATAGAC | QPCR |
| *CCD7a* RT-R | TCTGTGAAAGCCCAGTCAT | QPCR |
| *CCD7* RT-F | TGGTGAAGTTCGATACTGTG | QPCR |
| *CCD7* RT-R | CGTCGCTACCCTTTGATAC | QPCR |
| *CCD8* RT-F | CATTGTTGCCCGATCTGTT | QPCR |
| *CCD8* RT-R | GGCCAATCACCTTCCTCT | QPCR |
| *MAX2* RT-F | GCACATACTGCACCATC | QPCR |
| *MAX2* RT-R | GTAACGACAAACTCCTCTGG | QPCR |
| *BRC1* RT-F | CCCTTTTGGAGAGCATCAAG | QPCR |
| *BRC1* RT-R | AGACGTCGCGGATGAAGTAT | QPCR |
| *18S*-F | AAACGGCTACCACATCCAAG | QPCR |
| *18S*-R | ACTCGAAAGAGCCCGGTATT | QPCR |
| *Actin*-F | GACTGATGCGTTGATGAAGA | QPCR |
| *Actin*-R | TCATGAATACCAGCAGCT | QPCR |
