# Supplementary material for: Impacts of strigolactone on shoot branching under phosphate starvation in chrysanthemum (Dendranthema grandiflorum cv. Jinba)
Source: Front Plant Sci. 2015 Sep 11;6:694. doi: 10.3389/fpls.2015.00694 (PMC4566059; doi:10.3389/fpls.2015.00694)
Supplement: Supplementary file 4 [file Presentation1.PDF]

Supplementary Material

Impacts of Strigolactone on Shoot Branching under Phosphate Starvation in Chrysanthemum

Lin Xi[1], Chao Wen[1], Shuang Fang[2], Xiaoli Chen[1], Jing nie[1], JinFang Chu[2], Cunquan Yuan[1], Cunyu Yan 2[a §], Nan Ma[1 §], Liangjun Zhao [1 §*]

[1] Beijing Key Laboratory of Development and Quality Control of Ornamental Crops, Department of Ornamental Horticulture and Landscape Architecture, China Agricultural University, Beijing 100193, China
[2] National Centre for Plant Gene Research (Beijing), Institute of Genetics and Developmental Biology, Chinese Academy of Sciences, Beijing 100101, China
a. Current address: MIB & School of Chemistry, the University of Manchester, 131 Princess Street, Manchester M1 7DN, UK.
§ Both authors have contributed equally to the work
*** Correspondence:**
Liangjun Zhao, Beijing Key Laboratory of Development and Quality Control of Ornamental Crops, Department of Ornamental Horticulture and Landscape Architecture, China Agricultural University, Yuanmingyuan West Road, , Beijing 100193, China
zhaolj5073@sina.com

Supplementary Figure Legends

Supplementary Fig. S1. Split plate system, and schema of modified rooted nodal sections in node assay.
Schematic from left to right presented step-collection of nodal and rooted nodal section. Final rooted nodal section applying in split plate system was showed at very right. Root segments encompassed two nodes inserted between two agar-solidified media blocks in a petridish so that treatments could be applied apically or basally to the section. Red square marked detection node in experiment.

Supplementary Fig. S2. MRM-LC-MS/MS analysis of chrysanthemum (Dendranthema grandiflorum cv. Jinba) root exudates of plants grown under Pi starvation.
(A) Transitions 361>97; 363>97; 377>97 for root exudates.
(B) Full daughter ion scan MS/MS spectrum of three putative SL analogs in chrysanthemum exudate.

Supplementary Fig. S3. M^3 spectrogram of SLs from chrysanthemum
(A) 361>329>
(B) 363>331>
(C) 377>231>

Supplementary Fig. S4. Alignment, Phylogenetic tree and subcellular localization of characterizing gene DgCCD7(s)
(A) Alignment of the predicted amino acid sequences of DgCCD7 compared with Arabidopsis (MAX3), pea (RMS5), petunia (DAD7), and rice (D17). Intron positions corresponding to the genomic DNA sequence are denoted by black arrows.
(B) Phylogenetic analysis. Maximum likelihood (ML) phylogenetic tree was analyzed with 100 bootstrap pseudo replicates of CCD7 orthologues (Stamatakis, 2006) under the Dayhoff substitution matrix (Stamatakiset et al., 2008). *Actinidia chinensis* AcCCD7 (GU206813.1); *Arabidopsis thaliana* ArCCD7/MAX3 (NM_130064.4); *Artemisia annua* AaCCD7 (GQ468549.1); *Cucumis sativus* CsCCD7(HQ005419.1); *Dendranthema grandiflorum* DgCCD7a (KT004449) and DgCCD7b (KT004450); *Glycine max* GmCCD7 (NM_001254238.1); *Medicago truncatula* MtCCD7 (XM_003622507.1); *Petunia x hybrida* PhCCD7/DAD3 (FJ790878.1); *Pisum sativum* PsCCD7/ RMS5 (DQ403160.1); *Solanum lycopersicum* SlCCD7(GQ468556.1); *Orobanche ramosa* OrCCD7(JN412814.1); *Oryza sativa* OsCCD7/HTD1/D17(AL663000.4); *Vitis vinifera* VvCCD7 (XM_002274162.2); *Zea mays* ZmCCD7(NM_001196999.1).
(C) Subcellular localization of DgCCD7a using onion epidermal. Bar=50 μm.

Supplementary Fig. S5. Outgrowth activity of buds located different positions
Nodes from different positions (here we displayed 4 positions as representatives) were collected as one-node section. These sections were inserted into split plate system but only supplying regular MS medium apically and basally. Data were recorded every day and presented as means ± s.e.m (n= 15-20). Letters indicate significant differences among same positioned buds at α= 0.05.

Supplementary Figures

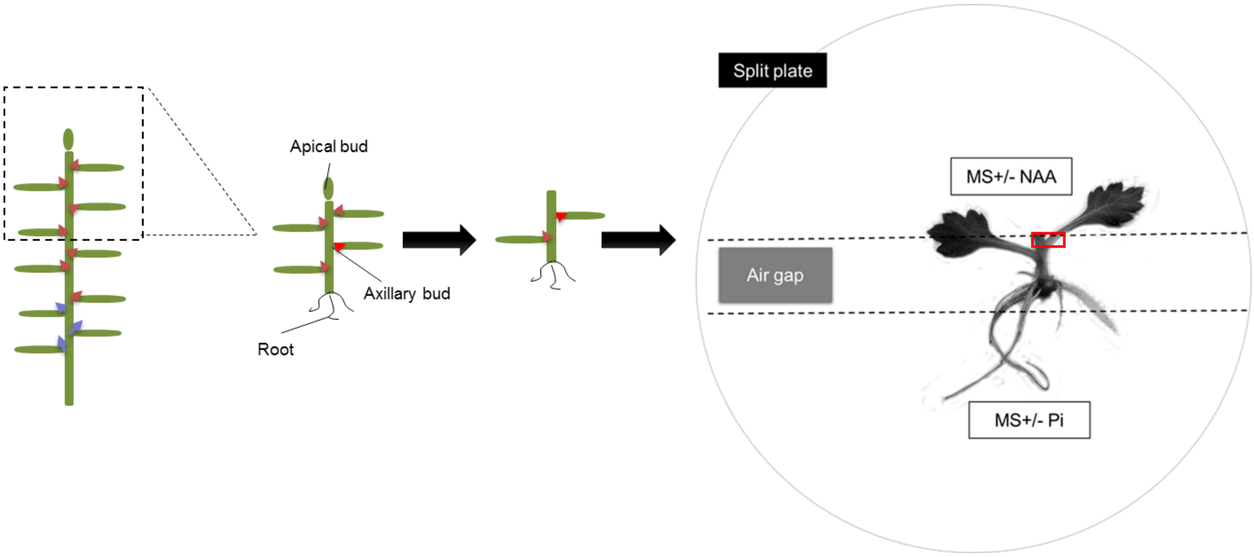

Supplementary Fig. S1.

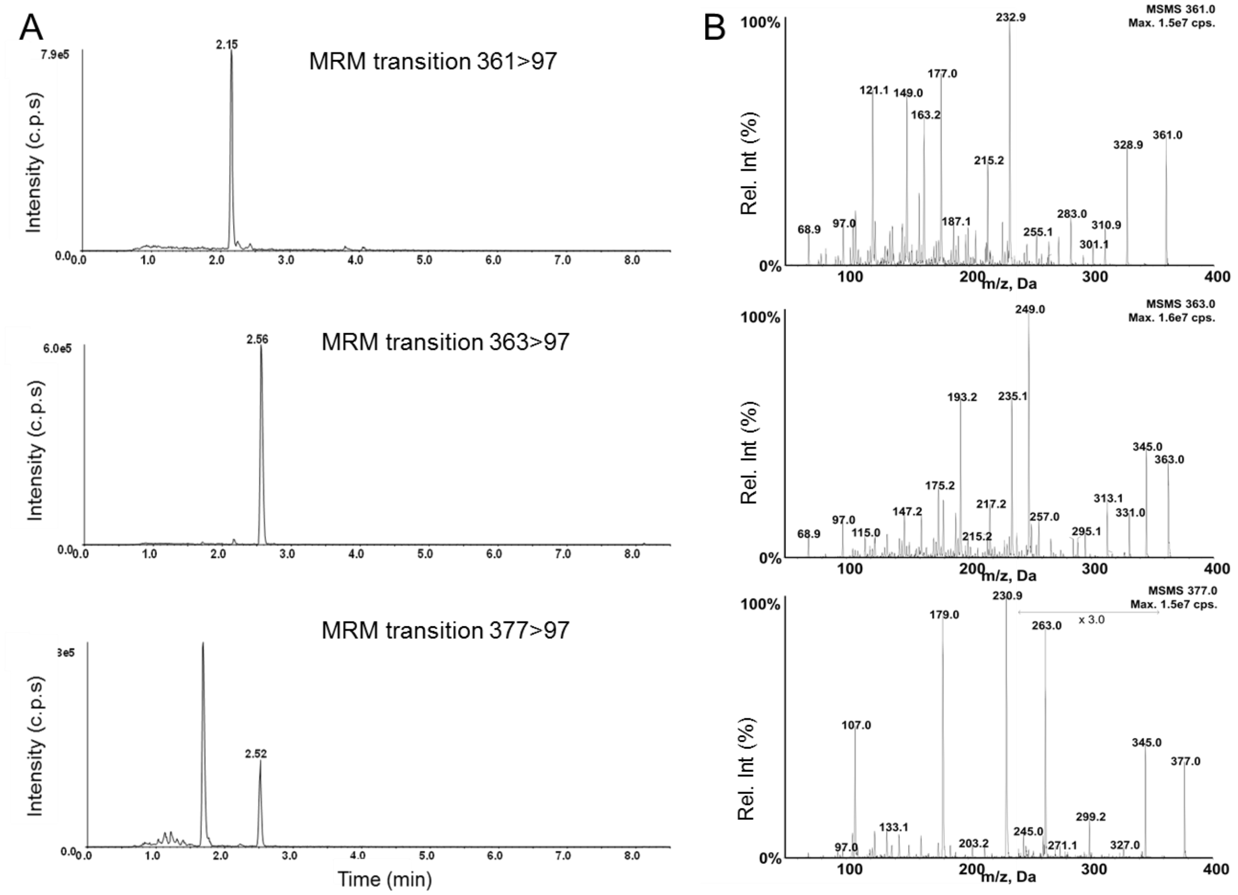

Supplementary Fig. S2.

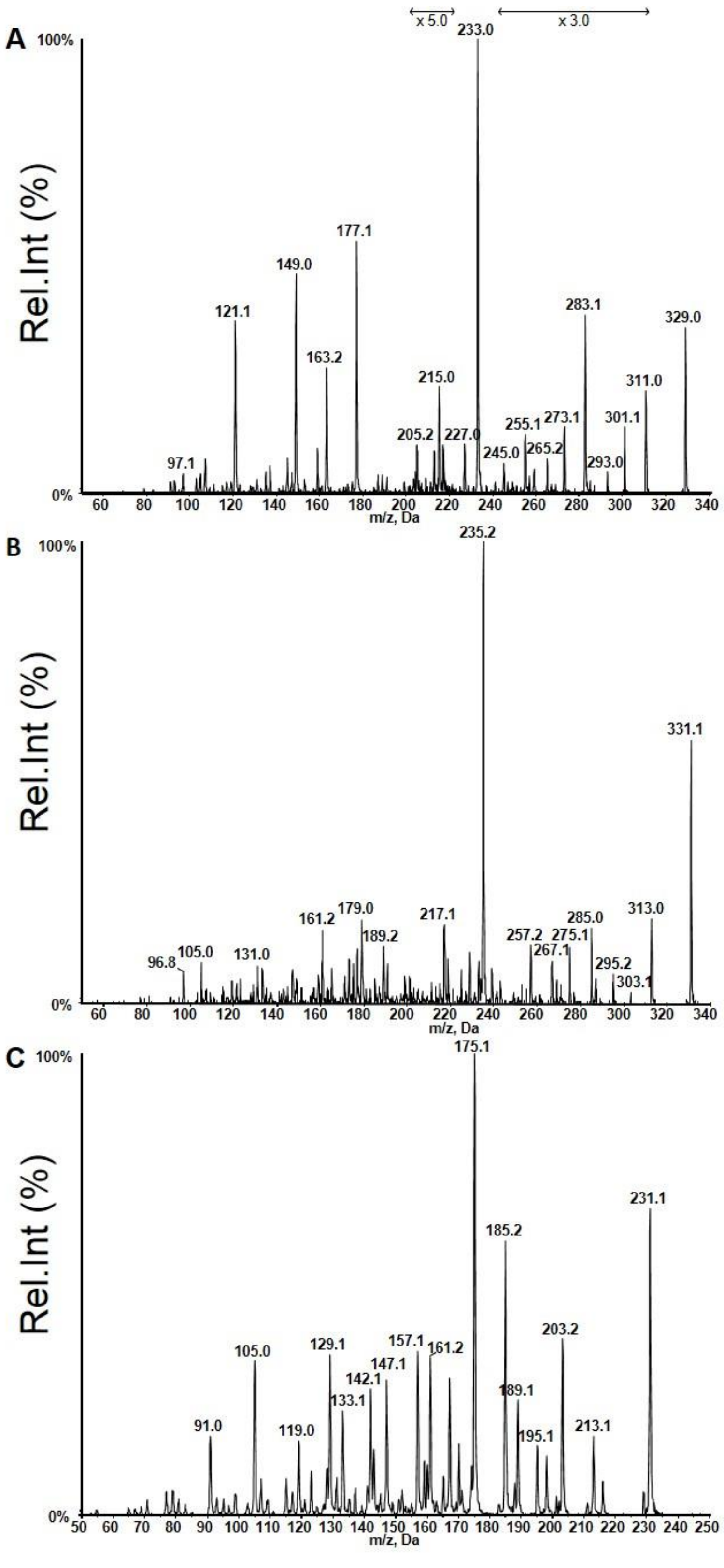

Supplementary Fig. S3.

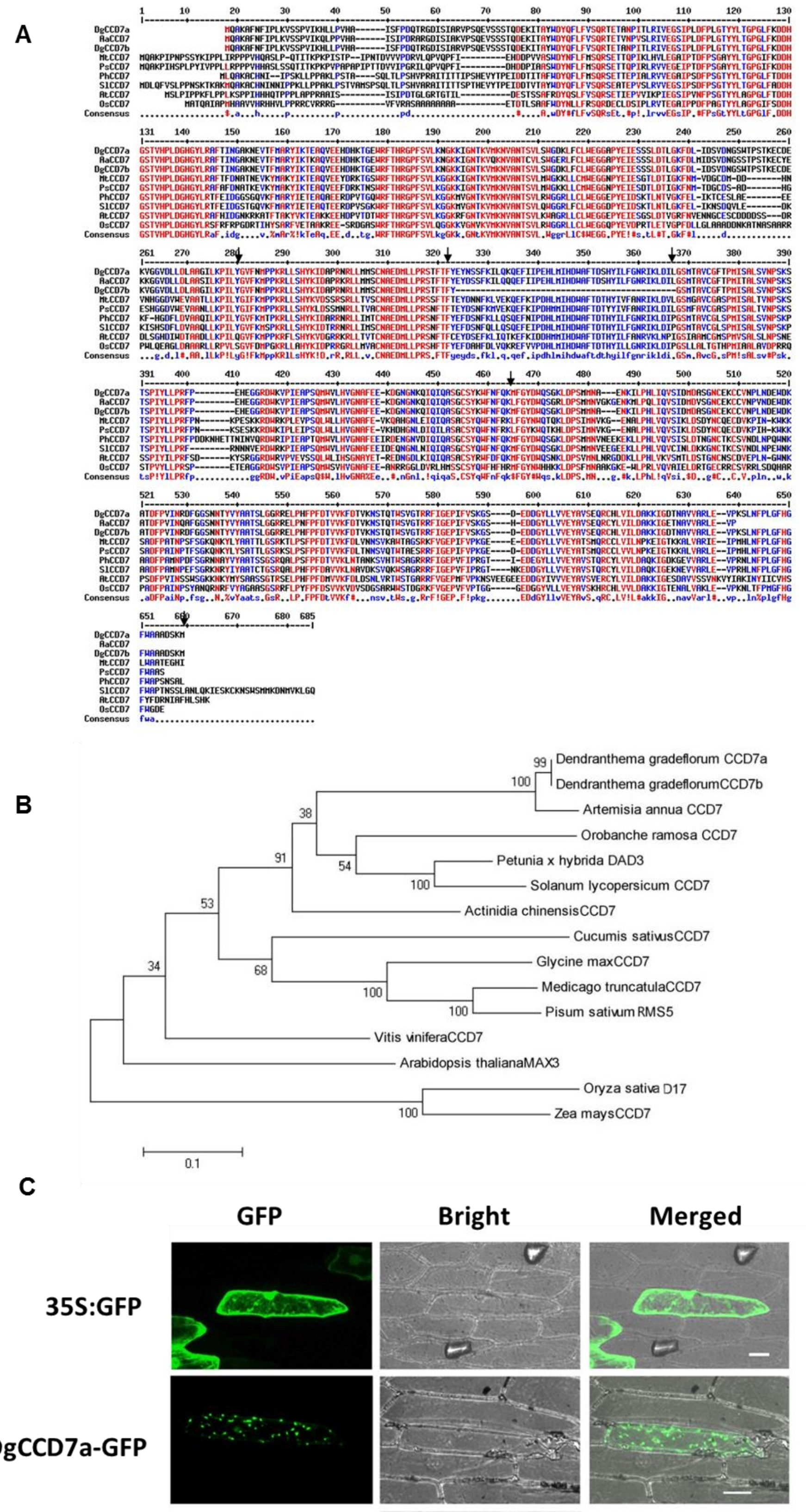

Supplementary Fig. S4.

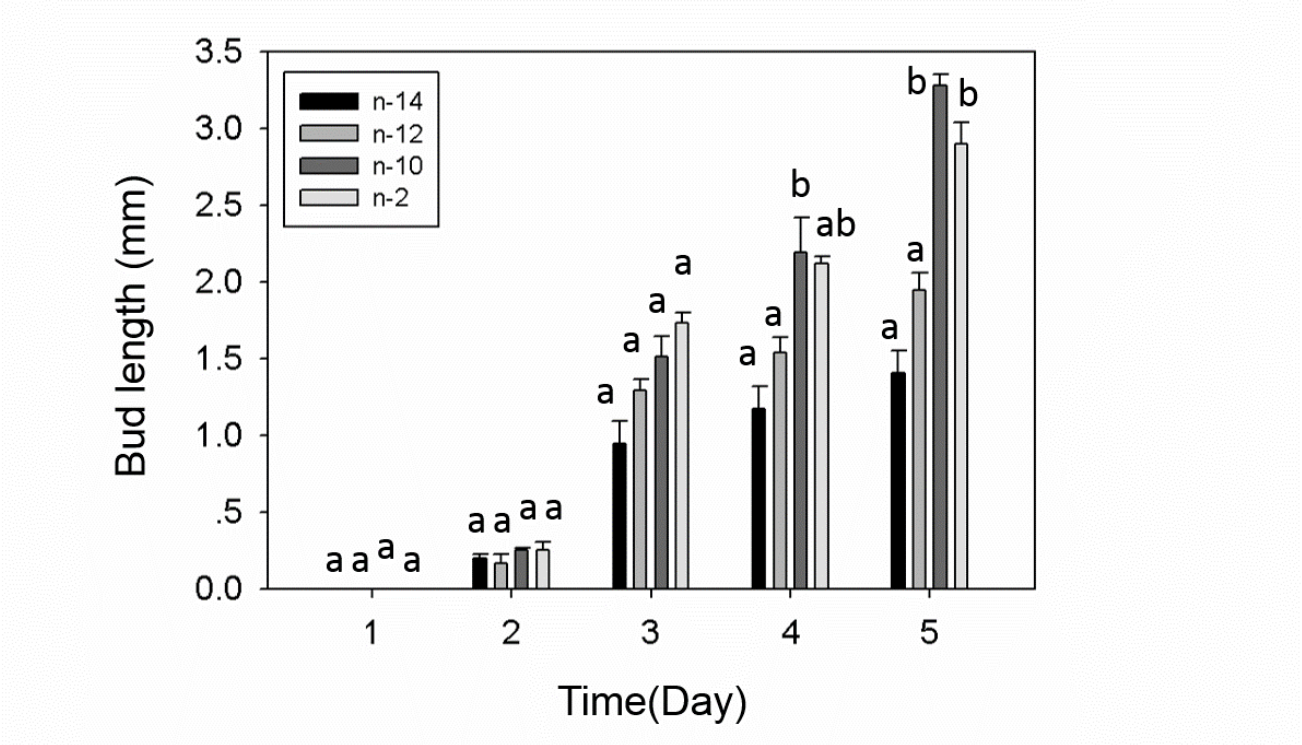

Supplementary Fig. S5.
